# Evaluating digital health literacy interventions for adults 45+ years: a scoping review

**DOI:** 10.1093/heapro/daag080

**Published:** 2026-06-09

**Authors:** Rachel Sinanan, George Van Doorn, Colette Browning, Britt Klein, Cameron Foale

**Affiliations:** Institute of Health and Wellbeing, Federation University, PO Box 663, Ballarat, VIC 3353, Australia; Institute of Health and Wellbeing, Federation University, PO Box 663, Ballarat, VIC 3353, Australia; Institute of Health and Wellbeing, Federation University, PO Box 663, Ballarat, VIC 3353, Australia; Institute of Health and Wellbeing, Federation University, PO Box 663, Ballarat, VIC 3353, Australia; Faculty of Business, Justice and Behavioural Sciences, School of Psychology, Charles Sturt University, 15 Blackall Street, Barton, ACT 2600, Australia; Institute of Health and Wellbeing, Federation University, PO Box 663, Ballarat, VIC 3353, Australia

**Keywords:** digital health literacy, health literacy, biopsychosocial, ageing

## Abstract

Digital health literacy is increasingly vital for equitable healthcare, yet adults aged 45+ may face age-related and psychosocial barriers that limit digital engagement. Interventions targeting these barriers are crucial. This review mapped the design and effectiveness of current digital health literacy interventions for adults 45+ years, assessing how they addressed biological, psychological, and social (biopsychosocial) dimensions and key outcomes. The review followed Joanna Briggs Institute methodology and Preferred Reporting Items for Systematic Reviews and Meta-Analyses extension for Scoping Reviews guidelines. Five databases were searched for English-language studies published between 2015 and 2025. Twenty-four studies met inclusion criteria, comprising randomized controlled trials, quasi-experimental studies, qualitative research, and systematic and scoping reviews. Data were synthesized and thematically mapped against biopsychosocial dimensions and effectiveness outcomes, including competence, critical thinking, empowerment, sustained engagement, and health outcomes. Most interventions were associated with improvements in digital skills, confidence, and self-efficacy, particularly when delivered face-to-face, in small groups, or through blended formats. However, most focused on psychological factors, with biological and social dimensions addressed inconsistently. Only one study incorporated all three biopsychosocial dimensions, and none assessed all effectiveness outcomes simultaneously. There was limited evidence for improvements in sustained engagement, critical thinking, and longer-term health outcomes following intervention. Digital health literacy interventions for adults aged 45+ years may therefore benefit from extending beyond a focus on technical skills to address the broader range of age-related biopsychosocial barriers. In this context, a biopsychosocial–digital framework appears to offer a more comprehensive foundation for the design and evaluation of interventions aimed at supporting meaningful and sustained health outcomes.

Contribution to Health Promotion

- Most available digital health programmes for adults 45+ focus only on basic technology skills.
- This review shows combining support for physical abilities, confidence, and social connections gives stronger, longer-lasting benefits.
- A holistic, person-centred approach helps people stay independent, access healthcare more easily, and age healthily.
- Findings can guide future research, practice, and policy to make digital health programmes more effective and inclusive.
- By addressing the whole person, not just technology, these programmes can better promote health, wellbeing, and equity for older adults.

## Digital health literacy in adults aged 45+ years: a scoping review

### Background

The rapid expansion of digital technologies, accelerated by the COVID-19 pandemic (Smuck *et al.* 2021, McBeath *et al.* 2022), has increased the need for older adults to develop competencies to navigate digital health environments. Adults aged 45 years and over may be more vulnerable to barriers to digital engagement and have lower health literacy than younger cohorts, partly due to age-related changes (e.g. biological factors, such as declines in vision; Shen *et al.* 2024). Consistent with this perspective, the present study adopts a 45+ years threshold aligned with the Organization for Economic Co-operation and Development (2025), which recognizes midlife (45–64 years) as a transitional period during which declines in health and digital health literacy (DHL) may begin (Levin-Zamir and Bertschi 2018, Jiang *et al.* 2024).

Health literacy is a multidimensional construct encompassing functional, interactive, and critical skills that enable effective engagement with health information and services (Nutbeam 2000, WHO 2022), alongside the knowledge, confidence, and self-efficacy required to access, understand, evaluate, remember, and apply health information (WHO 2022). Integrating both digital and health literacy, DHL is central to equitable and sustainable engagement with healthcare systems (Park *et al.* 2025). However, DHL is not simply the sum of these literacies. Rather, it is a context-specific skillset for navigating digital health environments, including engaging with platforms, interpreting multimodal information, evaluating credibility, and applying health knowledge in digital contexts. This conceptualization aligns with Norman and Skinner’s (2006) eHealth literacy model, which emphasizes multiple interacting literacies rather than a single skill. The scale of digital health use is substantial, with over 300 000 health apps currently in use globally, excluding wearables and other technologies (IQVIA Institute for Human Science 2024). As digital services proliferate, DHL has become increasingly important for healthcare access, self-management, and social connectedness, particularly among vulnerable populations (Cossu *et al.* 2025).

The biopsychosocial (BPS) framework conceptualizes health and behaviour as the product of interacting biological, psychological, and social factors, rather than any single factor acting in isolation (Engel 1977). This perspective has been widely applied across health research and practice, with evidence suggesting that BPS-informed approaches can improve health outcome evidence, suggesting that BPS-informed approaches can improve health outcomes (Kamper *et al.* 2015, Arthanat *et al.* 2018). Within the context of DHL, this framework provides a useful lens for understanding the multifaceted barriers faced by adults aged 45 years and over. Age-related changes in physical health (e.g. vision or cognitive functioning), psychological factors (e.g. motivation and self-efficacy), and social influences (e.g. support networks and access to resources) may interact to shape digital engagement. As such, barriers to DHL in mid-to-later life are unlikely to be adequately explained by deficits in technical skills alone and instead require consideration of broader BPS contexts (Arthanat *et al.* 2018).

Applying a BPS perspective to DHL also highlights the dynamic interplay between individual capacities and contextual influences. For example, interactions between cognitive ageing, confidence in using technology, and the availability of social support can influence how individuals engage with digital health information and services. Consistent with this, social and psychological factors have been shown to play a significant role in shaping older adults’ intentions to adopt and use digital technologies (Niu *et al.* 2026). However, existing DHL frameworks have been criticized for insufficiently accounting for contextual determinants, particularly in mid-to-later life, where structural and experiential factors may compound barriers to engagement (van der Vaart and Drossaert 2017, Sorensen *et al.* 2021). This suggests a need to more explicitly integrate BPS considerations into conceptualizations and interventions targeting DHL.

Importantly, DHL extends beyond foundational technical skills to encompass higher-order competencies, including the ability to critically evaluate information and apply it appropriately. That is, understanding not only how to access information but also why, when, and for whom it is relevant (Khan *et al.* 2022). These competencies are increasingly essential in digital environments characterized by large volumes of variable-quality information. Recent commentaries also highlight that while emerging tools such as AI-based systems (e.g. ChatGPT) may support access to health and nutrition information, they also place demands on users to critically appraise accuracy, relevance, and applicability (Arslan 2024, 2025). Together, these considerations reinforce the importance of conceptualizing DHL as a higher-order, context-dependent skillset embedded within the broader BPS framework, particularly for adults navigating the complexities of digital health in mid-to-later life. This perspective is especially pertinent given the interactions between psychological wellbeing and digital engagement, where factors such as motivation, cognitive load, and emotional distress can shape individuals’ capacity to effectively access, evaluate, and apply digital health information.

Globally, over 1 billion people experience mental illness, with rates of depression and anxiety increasing among older adults (WHO 2025). In Australia, the prevalence of mental illness among adults aged 35–54 years has also increased substantially (Australian Institute of Health and Welfare 2024), highlighting a growing psychosocial burden that may undermine motivation, confidence, cognitive capacity, and social participation (i.e. key determinants of DHL). As health systems increasingly rely on digital platforms for information access, service navigation, and self-management, older adults may face compounded barriers beyond technical skill deficits, including anxiety, reduced trust, and challenges in critically appraising online health information (Ahmadvand *et al.* 2018, Card 2023, Gyorffy *et al.* 2023). Together, these trends are directly relevant to DHL, as reduced motivation, increased cognitive load, and emotional distress can limit engagement with digital health information and services (O’Brien and Toms 2010). In addition, sociodemographic factors, such as age, education, socioeconomic status (SES), and digital access, are consistently associated with DHL, underscoring the importance of multidimensional, equity-focused interventions (van der Vaart and Drossaert 2017, Sørensen *et al.* 2021). These considerations highlight the need for interventions that address biological, psychological, and social factors alongside digital skills. Accordingly, this scoping review will map the range, characteristics, and outcomes of such interventions for adults aged 45+ years.

### Defining digital health literacy

Digital literacy was first defined as the ability to use digital technologies (Gilster 1997), but now includes critical, communicative, and participatory competencies for digital engagement (Ferrari 2013). Digital literacy has since been expanded to include the use of digital tools (e.g. telehealth) for health-related purposes, such as accessing information and supporting decision-making and behavioural change (Dunn and Hazzard 2019, Yoon *et al.* 2022, Australian Digital Health Agency 2025, Park *et al.* 2025). Importantly, digital literacy is a transferable foundational competency rather than a health-specific construct (Gilster 1997, Dunn and Hazzard 2019, Park *et al.* 2025).

DHL integrates digital and health literacy to support engagement with digital health systems (Zou *et al.* 2025). However, DHL is not an additive combination of these literacies, but a context-specific capability involving navigation of complex digital health environments, including interpreting information, engaging with services, and making decisions in contexts of uncertainty and variable information quality (Park *et al.* 2025, Zou *et al.* 2025). Makhafola *et al.* (2025) further distinguish DHL from digital competence, which involves more advanced technical skills (e.g. content creation and security), while DHL emphasizes communication, collaboration, critical thinking, and information evaluation.

Although definitions vary, most authors agree that DHL comprises confidence and problem-solving to locate, evaluate, and apply health information in digital contexts (Dunn and Hazzard 2019, WHO 2022, Yoon *et al.* 2022). This positions DHL as a higher-order, context-dependent literacy extending beyond operational digital skills to include critical appraisal and applied health decision-making (Dunn and Hazzard 2019, WHO 2022, Yoon *et al.* 2022). Collectively, these definitions reinforce the need to understand DHL within broader BPS contexts, rather than as a purely technical skill.

### Biopsychosocial factors influencing digital tool use in later life

To overcome barriers associated with ageing, understanding DHL for adults aged 45+ years requires attention to the broader BPS dimensions in which digital engagement occurs (Arthanat *et al.* 2018). Age-related changes can include working memory, vision, hearing, and dexterity, which can affect interactions with digital tools (Murman 2015, Salthouse 2019, Shams-Ghahfarokhi 2025). Psychologically, anxiety surrounding the use of digital technologies, fear of making mistakes, privacy concerns, and low self-efficacy can hinder engagement (Astell *et al.* 2020, Dykgraaf *et al.* 2022, Reid *et al.* 2024). Socially, limited support, isolation, intergenerational disconnects, and persistent age-based stereotypes reduce opportunities for meaningful digital participation (Kottl *et al.* 2021, Aleti *et al.* 2023, Xia and Chen 2025). These barriers emphasize the need to address the biological, psychological, and social limitations experienced more frequently by adults aged 45+ years.

Ahmadvand *et al.* (2018) and Card (2023) argue for extending the traditional BPS framework by incorporating a digital dimension, recognizing the influence of digital technologies, patient-generated data, and digital engagement. This BPS–digital model reflects the role of digital technologies as contextual factors that shape health behaviours, access to health information, social engagement, and identity (Ahmadvand *et al.* 2018). Within this framework, digital determinants interact with biological (e.g. sensory changes), psychological (e.g. confidence), and social factors (e.g. support networks), influencing middle-to-older aged adults’ engagement with digital health resources. Győrffy *et al.* (2023) argued that digital health encompasses not only technological change but also broader cultural and social transformation, including shifts in the doctor–patient relationship and health decision-making. Collectively, this emerging body of work supports the use of an expanded BPS framework to map the multidimensional factors relevant to DHL in middle-to-later life.

### Additional theoretical and conceptual frameworks supporting digital health literacy enhancement

Current theories supporting DHL interventions do not comprehensively address the BPS needs and barriers experienced by adults aged 45+ years. Commonly used frameworks, such as the Technology Acceptance Model (TAM; Davis 1989) or Information–Motivation–Behavioural Skills Theory (IMBS; Fisher *et al.* 2003), capture only the psychological and social dimensions of the BPS framework (Rahimi *et al.* 2018, Tsamlag *et al.* 2020). The core constructs of the TAM (i.e. perceived usefulness, perceived ease of use, attitudes towards technology, and behavioural intention) reflect cognitive, motivational, and affective processes central to the psychological domain, while extended versions incorporate social influences, such as subjective norms (Davis 1989). The IMBS model aligns primarily with psychological and social components of the BPS framework, emphasizing the personal and social motivation to engage in health-related behaviours (Fisher *et al.* 2003). However, both TAM and IMBS largely omit biological considerations, including age-related sensory, cognitive, and physical changes that may affect technology use, limiting their capacity to fully explain technological engagement among older adults.

### Current digital skills programmes addressing digital health literacy

Addressing the need for strong DHL is still in its early stages, particularly for vulnerable cohorts (e.g. older adults). Worldwide, the Global Digital Health Partnership (2025) is a collaboration of national government digital health authorities, and the WHO released modules and checklists, both with an aimed at enhancing engagement in digital health literacy. In Australia, community initiatives, such as Living Connected, aim to support older Australians in using digital devices, accessing My Health Record, and engaging safely online (Australian Digital Health Agency 2025). Similarly, the Be Connected platform delivers digital skills training to Australians aged 50+ years, although programme availability remains uneven, with face-to-face training limited to certain regions (Be Connected n.d.). However, these initiatives often prioritize basic technical skills over higher-order competencies, such as critical evaluation and the application of health-related information, thereby providing foundational digital skills rather than comprehensive DHL (Be Connected n.d., Hasan and Linger 2016). This gap is particularly concerning given the increasing prevalence of psychosocial disabilities, including long-term mental health conditions that can limit social participation, among adults aged 45+ years (Reed 2024, WHO 2024).

### Purpose and rationale of the scoping review

Reviews of DHL interventions for older adults suggest that approaches such as personalized tutoring, peer learning, intergenerational support, and carefully paced instruction can help address cognitive, social, and psychological needs (Xu *et al.* 2025). However, the evidence remains fragmented, with limited clarity regarding which interventions are most effective and sustainable. Despite the growing body of research, important gaps remain in understanding how DHL interventions address the intersecting biological, psychological, social, and digital needs of adults aged 45+ years.

Given the accelerating pace of digital change, the diversity of skills and needs within those aged 45+ years, and the implications for equitable access to healthcare, this scoping review aims to map the extent to which DHL interventions address BPS needs and contribute to meaningful outcomes. Specifically, the review addresses two questions:

1. To what extent do DHL interventions address the biological, psychological, and social needs of adults aged 45+ years?
2. How effective are these interventions in enhancing competence, critical thinking, empowerment, sustained engagement, and health outcomes?

By synthesizing evidence across disciplines, this review seeks to clarify the effectiveness, inclusiveness, and person-centeredness of DHL interventions for adults aged 45+ years, thereby providing a foundation for future research and informing the development of practical, evidence-based solutions.

## Methods

### Transparency and openness statement

The authors declare their commitment to promoting transparency and openness in research. A protocol was developed to guide the systematic mapping of key concepts and evidence, with a focus on determining whether DHL interventions for adults aged 45+ years address BPS needs and whether they enhance competence, critical thinking, empowerment, sustained engagement, and health outcomes. The protocol can be accessed via the Open Science Framework at https://osf.io/qnv9e/overview (registration number QNV9E). This protocol was developed prior to the commencement of data extraction, and any deviations from this protocol are explicitly outlined in the Methods section. We confirm adherence to the PRISMA-ScR (Preferred Reporting Items for Systematic Reviews and Meta-Analyses extension for Scoping Reviews; Tricco *et al.* 2018) reporting guidelines, and the scoping review was conducted in accordance with the Joanna Briggs Institute (JBI) methodology (Peters *et al.* 2024).

All data extraction forms and the final synthesized dataset (stripped of any identifying or confidential information) are available publicly via Open Science Framework (https://dx.doi.org/10.17605/OSF.IO/498GM). All funding sources are acknowledged in the Funding section, and the authors have declared no conflicts of interest. No primary data were collected, generated, or analysed. All data included in this review were derived from publicly available sources, and the search strategy, eligibility criteria, screening procedures, and data charting processes are described in detail in the Methods section to support transparency and reproducibility. Accordingly, there are no de-identified datasets associated with this manuscript. As the study did not involve statistical modelling or computational analyses, no analytic code was generated. Further, because this was a review of existing literature and did not involve the development of original experimental materials, survey instruments, or intervention content, there are no study materials to make available beyond those described in the manuscript.

### Ethical approval

This review is a secondary analysis of existing data. Therefore, ethical approval was not required. All data used were from published, peer-reviewed, or publicly available sources and have been cited appropriately.

### Data sources and search strategy

The search strategy was designed to identify published, peer-reviewed studies relevant to the review objectives. A three-step search strategy was employed. First, an initial exploratory search of Web of Science, PubMed, PsycINFO, Cochrane, and EBSCO was conducted to locate key articles on the topic. Next, the text words contained in the titles and abstracts of these articles, along with their index terms, were analysed to refine and develop a comprehensive search strategy. Then, the final search strategy was adapted for each database and information source, incorporating all relevant keywords and vocabulary terms. Search terms were developed to capture four key domains: digital health literacy, BPS context, target population, and outcomes. Specifically, terms encompassed concepts related to DHL, the BPS model, adults aged 45+ years, and skills and competence. These terms were combined using Boolean operators to form the final search string: (‘digital literacy’ OR ‘eHealth literacy’ OR ‘digital health literacy’ OR ‘health information literacy’) AND (‘biopsychosocial’ OR ‘holistic’ OR ‘psychosocial’ OR ‘social determinants’ OR ‘person-centred’ OR ‘patient-centered’ OR ‘integrated care’) AND (‘training’ OR ‘intervention*’ OR ‘program*’ OR ‘education’ OR ‘course*’ OR ‘assessment’) AND (‘older adult*’ OR ‘elder*’ OR ‘senior*’ OR ‘geriatric*’ OR ‘aging population’ OR ‘ageing population’ or aged 45+’) AND (‘competence’ OR ‘skills’ OR ‘critical thinking’ OR ‘empowerment’ OR ‘self-efficacy’ OR ‘sustained engagement’ OR ‘health outcome*’)

To ensure comprehensive coverage, the reference lists of included sources were also screened for additional eligible publications. The review considered a broad range of study designs. Eligible designs included experimental and quasi-experimental study designs [randomized controlled trials (RCTs), non-RCTs], before and after studies, and interrupted time-series analyses. Analytical observational studies, such as prospective and retrospective cohort studies, case–control studies, and analytical cross-sectional studies, were also included. Systematic reviews, descriptive observational studies (case series, case reports), and descriptive cross-sectional studies were likewise eligible for inclusion.

### Study selection criteria

The selection criteria were designed to prioritize contemporary evidence, with a particular focus on developments over the past decade. This timeframe was chosen to reflect the rapid increase of digital health technologies and heightened demand for DHL support following the COVID-19 pandemic (Choukou *et al.* 2022). Studies were included if they were published in English between November 2015 and November 2025, involved adults aged 45 years and over, and examined interventions targeting digital or electronic health literacy with reported outcomes. Studies were excluded if they were not published in English, did not focus on participants aged 45+ years, or did not evaluate an intervention aimed at improving DHL or electronic health literacy skills.

### Data extraction, critical appraisals, and synthesis

**Figure 1:**
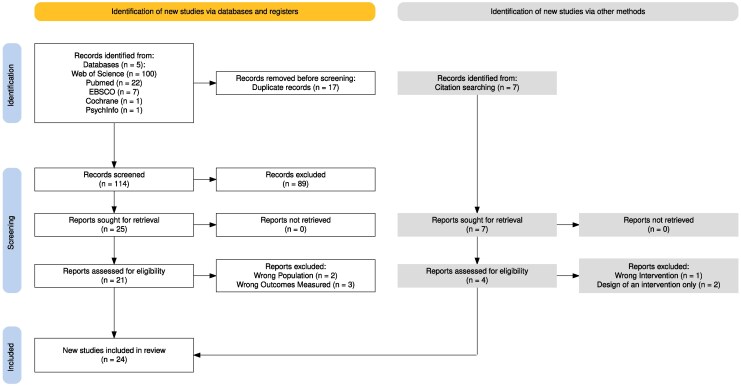
PRISMA diagram.

Following the search, all identified citations were imported into Endnote 21 (Endnote 2025), and duplicates were removed. The remaining records were exported to an Excel spreadsheet, where two reviewers (R.S. and G.V.D.) independently screened titles and abstracts against the predefined inclusion criteria. Any discrepancies were resolved through discussion. Full texts of potentially relevant studies were then retrieved and assessed in detail using Covidence systematic review software (Covidence 2025), with the same two reviewers applying the inclusion criteria. Reasons for exclusion of full-text articles were documented and are presented in the PRISMA flow diagram (see Fig. 1; Page *et al.* 2021).

Data extracted captured key information regarding participants, key concepts, context, study design and methods, and findings relevant to the review questions. The extracted data were synthesized descriptively, with full study characteristics reported in the Characteristics Table (Supplementary Table S1) and key outcomes mapped to the review questions in Tables 1 and 2. To minimize duplication, findings from primary studies and review-level evidence were clearly distinguished during synthesis.

**Table 1:** Interventions addressing biological, psychological, and social domains.

Key findings	Specific findings	Articles
Biological	Consideration of age-related changes in ability to navigate digital tools when designing and training, for ease of use	Ahmad and Mozelius (2019), Chang *et al.* (2021a), De Main *et al.* (2022), Fink and Beck (2015), Li *et al.* (2021), Ngiam *et al.* (2022), Vaswani *et al.* (2023), Ghorbanian Zolbin *et al.* (2022)
	Tutorial interventions giving consideration to biological barriers	Ahmad and Mozelius (2019), Chang *et al.* (2021a), Li *et al.* (2021), Ngiam *et al.* (2022), Vaswani *et al.* (2023), Ghorbanian Zolbin *et al.* (2022)
Psychological	Trust in digital health applications and personal integrity within the applications (e.g. addressing privacy issues)	Ahmad and Mozelius (2019), He *et al.* (2025), Malone *et al.* (2017), Nahm *et al.* (2019), Yang *et al.* (2024), Zhang *et al.* (2025)
	Technology acceptance and perceived usefulness	Ahmad and Mozelius (2019), Bevilacqua *et al.* (2021), Chang *et al.* (2021b), de Guzman and Dino (2020), De Main *et al.* (2022), Fink and Beck (2015), Goransson *et al.* (2020), He *et al.* (2025), Lee and Kim (2019), Miller *et al.* (2024), Nahm *et al.* (2019), Ngiam *et al.* (2022), Pourrazavi *et al.* (2020), Vaswani *et al.* (2023), Vazquez *et al.* (2023), Yang *et al.* (2024), Zhang *et al.* (2025), Ghorbanian Zolbin *et al.* (2022)
	Self-efficacy	Banbury *et al.* (2020), Banbury *et al.* (2020), Chang *et al.* (2021a, 2021b), de Guzman and Dino (2020), Fink and Beck (2015), He *et al.* (2025), Li *et al.* (2021), Miller *et al.* (2024), Nahm *et al.* (2019), Ngiam *et al.* (2022), Pourrazavi *et al.* (2020), Vazquez *et al.* (2023), Yameogo *et al.* (2025), Yang *et al.* (2024), Zhang *et al.* (2025), Ghorbanian Zolbin *et al.* (2022)
	Motivation to enact behavioural change	Bevilacqua *et al.* (2021), Chang *et al.* (2021a, 2021b), de Guzman and Dino (2020), Fink and Beck (2015), Goransson *et al.* (2020), Lee and Kim (2019), Pourrazavi *et al.* (2020), Vazquez *et al.* (2023), Wang and Luan (2022), Yameogo *et al.* (2025)
Social	Social connectedness during training and usage	Banbury *et al.* (2020), He *et al.* (2025), Lee and Kim (2019), Miller *et al.* (2024), Pourrazavi *et al.* (2020), Vazquez *et al.* (2023), Wang and Luan (2022), Yameogo *et al.* (2025), Yang *et al.* (2024), Zhang *et al.* (2025)
	Perceived feeling of support	Banbury *et al.* (2020), Chang *et al.* (2021a), Goransson *et al.* (2020), Lee and Kim (2019), Malone *et al.* (2017), Miller *et al.* (2024), Wang and Luan (2022), Yameogo *et al.* (2025), Yang *et al.* (2024), Zhang *et al.* (2025)

**Table 2:** Effectiveness outcomes.

Key outcomes	Specific outcomes	Articles
Competence	Codesign enhanced competence and usability	Ahmad and Mozelius (2019), Banbury *et al.* (2020), Bevilacqua *et al.* (2021), De Main *et al.* (2022), Goransson *et al.* (2020), Lee and Kim (2019), Li *et al.* (2021), Pourrazavi *et al.* (2020), Vaswani *et al.* (2023), Yang *et al.* (2024)
	Self-belief: confidence is crucial for competency	Ahmad and Mozelius (2019), Banbury *et al.* (2020), Bevilacqua *et al.* (2021), Chang *et al.* (2021a, 2021b), de Guzman and Dino (2020), De Main *et al.* (2022), Goransson *et al.* (2020), Lee and Kim (2019), Li *et al.* (2021), Pourrazavi *et al.* (2020), Vaswani *et al.* (2023), Yameogo *et al.* (2025), Yang *et al.* (2024)
	Intergenerational, peer, or group training enhances competence	Bevilacqua *et al.* (2021), Chang *et al.* (2021b), De Main *et al.* (2022), He *et al.* (2025), Lee and Kim (2019), Malone *et al.* (2017), Miller *et al.* (2024), Ngiam *et al.* (2022), Pourrazavi *et al.* (2020), Vaswani *et al.* (2023), Vazquez *et al.* (2023), Yameogo *et al.* (2025), Yang *et al.* (2024), Zhang *et al.* (2025), Ghorbanian Zolbin *et al.* (2022)
Critical thinking	Ability to source and understand credible information	Chang *et al.* (2021a, 2021b), De Main *et al.* (2022), Malone *et al.* (2017), Yang *et al.* (2024), Ghorbanian Zolbin *et al.* (2022)
Empowerment	Training for independence	Ahmad and Mozelius (2019), Banbury *et al.* (2020), Chang *et al.* (2021a), De Main *et al.* (2022), Fink and Beck (2015), Goransson *et al.* (2020), He *et al.* (2025), Lee and Kim (2019), Malone *et al.* (2017), Miller *et al.* (2024), Ngiam *et al.* (2022), Pourrazavi *et al.* (2020), Vaswani *et al.* (2023), Wang and Luan (2022), Yameogo *et al.* (2025), Zhang *et al.* (2025), Ghorbanian Zolbin *et al.* (2022)
	Intergenerational, multimedia, and group learning enhances empowerment	Bevilacqua *et al.* (2021), Chang *et al.* (2021b), De Main *et al.* (2022), Goransson *et al.* (2020), He *et al.* (2025), Lee and Kim (2019), Malone *et al.* (2017), Miller *et al.* (2024), Ngiam *et al.* (2022), Pourrazavi *et al.* (2020), Vaswani *et al.* (2023), Vazquez *et al.* (2023), Yameogo *et al.* (2025), Yang *et al.* (2024), Zhang *et al.* (2025)
Sustained engagement	Ongoing behavioural change	Chang *et al.* (2021b), de Guzman and Dino (2020), Lee and Kim (2019), Miller *et al.* (2024), Wang and Luan (2022), Yameogo *et al.* (2025), Yang *et al.* (2024)
	Enabled access to digital tools	Chang *et al.* (2021b), Fink and Beck (2015), Wang and Luan (2022), Yang *et al.* (2024)
Positive health outcomes	DHL creates: training increases use of digital tools and leads to improved health outcomes	Banbury *et al.* (2020), Bevilacqua *et al.* (2021), Chang *et al.* (2021a), de Guzman and Dino (2020), Fink and Beck (2015), Goransson *et al.* (2020), He *et al.* (2025), Ngiam *et al.* (2022), Pourrazavi *et al.* 2020, Vazquez *et al.* (2023), Wang and Luan (2022), Zhang *et al.* (2025)

Although critical appraisal is not mandatory for scoping reviews (Roga and Pruis 2025), the authors deemed it important for evaluating the trustworthiness, relevance, and contribution of research in this emerging area. Consistent with the JBI methodology (Peters *et al.* 2024), appropriate critical appraisal tools were applied according to study design. Systematic and scoping reviews (*n* = *7*) were appraised using the Aromataris *et al.* (2015) tool, RCTs (*n* = *3*) were assessed using the Barker *et al.* (2023) tool, quasi-experimental studies (*n* = *12*) were appraised using the Barker *et al.* (2024) tool, and qualitative studies (*n* = *2*) were evaluated using the Lockwood *et al.* (2015) tool. Full risk-of-bias assessments are provided in Supplementary Appendix S1. Critical appraisal was not used as a basis for study exclusion. Rather, appraisal ratings informed the interpretation of findings by indicating the level of confidence placed in reported outcomes.

NVivo software (version 15.3.1; Lumivero 2025) was used to support synthesis of the finding. For review question one, it facilitated systematic coding of biological, psychological, and social dimensions. For research question two, it was used to organize and analyse qualitative data relating to competence, critical thinking, empowerment, sustained engagement, and health outcomes. This approach enhanced transparency and facilitated comprehensive mapping of findings, with results presented in Appendices S2 and S3.

## Results

The initial database search identified 131 articles, including 100 from Web of Science, 22 from PubMed, seven from EBSCO, one from Cochrane, and one from PsycINFO. After removal of 17 duplicates, 114 titles and abstracts were screened. This process resulted in 24 articles being retrieved for full-text review. An additional seven records were identified through manual inspection of reference lists.

Following full-text assessment, two articles were excluded due to an irrelevant study population, three measured outcomes outside of the scope of the review, two were removed for lacking an intervention component, and one for employing an ineligible intervention type. Consequently, 24 studies met the inclusion criteria. A PRISMA flowchart (generated using the Shiny app for PRISMA 2020 compliant flowcharts; Haddaway *et al.* 2022) summarized the search and selection process (see Fig. 1).

### Characteristics of the included studies

Across the 24 studies, interventions were most commonly delivered in senior or healthcare settings, with others implemented in libraries, participants’ homes, or fully online. Review articles typically did not specify a setting. Methodologically, the evidence base was dominated by quasi-experimental designs and narrative or systematic reviews, with comparatively few RCTs or qualitative investigations. Participants were consistently described as older adults, although the ages of included participants varied substantially, ranging from 47+ years to 74+ years.

DHL interventions for adults aged 45+ years were most commonly informed by behavioural/cognitive theories (e.g. TAM and Health Belief Model) and educational models (e.g. andragogy and multimedia learning). Fewer studies applied multilevel or ageing-specific frameworks, such as social ecological frameworks, the Digital Competence Framework for Citizens, or the European Commission framework defining five core areas, including information literacy, communication, content creation, safety, and problem-solving (Vuorikari *et al.* 2022). Notably, several primary studies did not report any explicit theoretical underpinning. The full table presented the characteristics of the included studies, including the first authors, location, study design, setting, population, age, year of publication, and theoretical frameworks (Supplementary Table S1).

### Measurements, intervention type, and results

#### Measurements

DHL outcomes were assessed using a diverse range of validated instruments, performance-based assessments, and qualitative methods. The eHealth Literacy Scale (eHEALS; Norman and Skinner 2006) was the most used instrument (nine studies). Broader health literacy measures, technology-acceptance measures, and digital-confidence scales appeared less often, while objective skill-based assessments (e.g. information-seeking tasks or website evaluation exercises) were reported in only a minority of studies. Psychosocial constructs, such as self-efficacy, social support, and loneliness, were commonly examined as secondary outcomes. Two reviews note inconsistency in measurement approaches, including variation in the use of validated instruments across the evidence base (Wang and Luan 2022, Yang *et al.* 2024).

#### Intervention types

Interventions varied based on delivery mode. Face-to-face training was most prevalent (Malone *et al.* 2017, Lee and Kim 2019, Bevilacque *et al.* 2020, de Guzman and Diño 2020, Chang *et al.* 2021a, Miller *et al.* 2024, He *et al.* 2025, Yameogo *et al.* 2025), typically delivered in community or home settings and emphasizing hands-on practice and personalized support. Online programmes commonly used videoconferencing, web-based modules, and app-based learning, often supplemented with structured follow-up (Banbury *et al.* 2019, Goransson *et al.* 2020, Li *et al.* 2021, de Main *et al.* 2022, Ngiam *et al.* 2022, Vaswani *et al.* 2023). Intergenerational or peer-mentoring approaches were associated with added benefits, particularly in building confidence and social connectedness (Lee and Kim 2019, Chang *et al.* 2021a, Miller *et al.* 2024, Yameogo *et al.* 2025). Blended approaches, which integrated in-person training with complementary digital resources, indicated that participants were able to consolidate skills through both guided instruction and independent practice. Review articles consistently identified education, guided practice, usability support, and tailored assistance as core components of effective interventions while also highlighting persistent challenges related to access, digital infrastructure, and long-term sustainability.

#### Intervention outcomes

Across the 24 studies included in this review, most interventions reported improvements in eHealth literacy, digital skills, attitudes towards technology, and psychosocial outcomes, although effect sizes varied according to delivery mode and intervention intensity. Face-to-face, blended, and small-group programmes incorporating hands-on practice were associated with consistent and sustained gains (Lee and Kim 2019, Nahm *et al.* 2019, Banbury *et al.* 2020, Bevilacqua *et al.* 2021, Chang *et al.* 2021a, Ngiam *et al.* 2022, Vazquez *et al.* 2023, Miller *et al.* 2024), though some studies reported attenuation of effects over time (Vazquez *et al.* 2023. Mentoring and socially supported approaches were associated with improvements in both digital literacy and wellbeing (Lee and Kim 2019, Miller *et al.* 2024, He *et al.* 2025, Yameogo *et al.* 2025). Online and videoconferencing interventions were associated with statistically significant but variable improvements, while low-intensity or exclusively digital approaches were associated with smaller or nonsignificant effects (Banbury *et al.* 2020, Göransson *et al.* 2020, He *et al.* 2025). Qualitative findings underscored the importance of trust, privacy, and social support as key facilitators of engagement (Ahmad and Mozelius 2019, Vaswani *et al.* 2023, Yameogo *et al.* 2025). Supplementary Table S2 summarizes the measurements, intervention types, and outcomes reported across the included studies.

### Interventions addressing biological, psychological, and social dimensions

Table 1 summarizes the extent to which interventions address each BPS domain. Only one study addressed all three BPS dimensions simultaneously (Chang *et al.* 2021b), and although it reported significant improvements in DHL, the sample size was small (*N* = 11), limiting generalizability.

#### Biological

Included studies consistently accounted for age-related functional changes when designing and delivering digital health interventions, with a strong emphasis on usability and accessibility to support ease of navigation. These biological considerations were evident in interface design choices and in the provision of tailored tutorials or training intended to accommodate sensory, cognitive, and motor limitations associated with ageing (Fink and Beck 2015, Ahmad and Mozelius 2019, Li *et al.* 2021, Chang *et al.* 2021b, De Main *et al.* 2022, Ghorbanian Zolbin *et al.* 2022, Ngiam *et al.* 2022, Vaswani *et al.* 2023). Overall, biological dimensions represented 16% of all coded intervention themes (Supplementary Figure S1 and Appendix S2).

#### Psychological

Psychological factors were the most frequently addressed domain. Studies highlighted trust in digital health interventions, perceived integrity, technology acceptance, and perceived usefulness as central determinants of engagement. Interventions that targeted confidence, motivation, and attitudes towards technology use were often associated with improvements in self-efficacy and behavioural change (Ahmad and Mozelius 2019, Lee and Kim 2019, Nahm *et al.* 2019, de Guzman and Dino 2020, Goransson *et al.* 2020, Pourrazavi *et al.* 2020, Bevilacqua *et al.* 2021, Chang *et al.* 2021a, 2021b, De Main *et al.* 2022, Ghorbanian Zolbin *et al.* 2022, Ngiam *et al.* 2022, Vaswani *et al.* 2023, Vazquez *et al.* 2023, Miller *et al.* 2024, Yang *et al.* 2024, He *et al.* 2025, Yamegeo *et al.* 2025, Zhang *et al.* 2025). Psychological dimensions accounted for 57% of all coded intervention themes (Supplementary Figure S1 and Appendix S2).

#### Social

Social dimensions were addressed through strategies that fostered social connectedness and perceived support during training and use of digital health tools. Group-based, peer-supported, or facilitator-led approaches were frequently reported and were associated with improved engagement and sustained use, aligning with emerging evidence (Niu *et al.* 2026). Perceived social support was consistently and positively associated with participation and confidence in and ongoing use of digital tools (Malone *et al.* 2017, Lee and Kim 2019, Banbury *et al.* 2020, Goransson *et al.* 2020, Chang *et al.* 2021b, Ghorbanian Zolbin *et al.* 2022, Wang and Luan 2022, Vazquez *et al.* 2023, Miller *et al.* 2024, Yang *et al.* 2024, He *et al.* 2025, Yameogo *et al.* 2025, Zhang *et al.* 2025). Social dimensions represented 27% of all coded intervention themes (Supplementary Figure S1 and Appendix S2).

### Effectiveness outcomes

Table 2 summarizes the effectiveness outcomes reported across the studies. While individual studies assessed specific effectiveness outcomes, no single study addressed all five DHL effectiveness domains (i.e. competency, critical thinking, empowerment, sustained engagement, and positive health outcomes). Given the heterogeneity of study designs and methodological limitations associated with the included studies, findings should be interpreted as indicative rather than conclusive evidence of intervention effectiveness.

#### Competence

Improvements in digital competence were commonly reported, particularly in interventions that incorporated codesign principles (Ahmad and Mozelius 2019, Lee and Kim 2019, Banbury *et al.* 2020, Goransson *et al.* 2020, Pourrazavi *et al.* 2020, Bevilacqua *et al.* 2021, Li *et al.* 2021, De Main *et al.* 2022, Vaswani *et al.* 2023, Yang *et al.* 2024). Increased DHL competence was frequently associated with enhanced self-belief, confidence, and intergenerational and peer- and group-based training approaches (Malone *et al.* 2017, Ahmad and Mozelius 2019, Banbury *et al.* 2020, de Guzman and Dino 2020, Bevilacqua *et al.* 2021, Chang *et al.* 2021a, De Main *et al.* 2022, Ghorbanian Zolbin *et al.* 2022, Ngiam *et al.* 2022, Vazquez *et al.* 2023, Miller *et al.* 2024, He *et al.* 2025, Yameogo *et al.* 2025, Zhang *et al.* 2025). Competence outcomes accounted for 28% of coded effectiveness themes (Supplementary Figure S2 and Appendix S3).

#### Critical thinking

Several studies reported improvements in participants’ ability to locate, evaluate, and interpret credible digital information following intervention participation (Malone *et al.* 2017, Chang *et al.* 2021a, 2021b, De Main *et al.* 2022, Ghorbanian Zolbin *et al.* 2022, Yang *et al.* 2024). These improvements were associated with more informed and deliberate engagement with digital health information. Critical thinking outcomes represented 7% of coded effectiveness themes (Supplementary Figure S2 and Appendix S3).

#### Empowerment

Many interventions aimed to enhance participants’ independence, with studies reporting increased autonomy and perceived control over digital technology use (Malone *et al.* 2017, Ahmad and Mozelius 2019, Lee and Kim 2019, Banbury *et al.* 2020, Goransson *et al.* 2020, Chang *et al.* 2021b, De Main *et al.* 2022, Ghorbanian Zolbin *et al.* 2022, Nahm *et al.* 2022, Wang and Luan 2022, Miller *et al.* 2024, He *et al.* 2025, Yameogo *et al.* 2025, Zhang *et al.* 2025). Intergenerational, multimedia, and group-based learning approaches were frequently identified as key mechanisms underpinning these gains, supporting empowerment through both skill development and social interaction (Malone *et al.* 2017, Lee and Kim 2019, Goransson *et al.* 2020, Pourrazavi *et al.* 2020, Bevilacqua *et al.* 2021, Chang *et al.* 2021a, De Main *et al.* 2022, Ngiam *et al.* 2022, Vaswani *et al.* 2023, Vazquez *et al.* 2023, Miller *et al.* 2024, Yang *et al.* 2024, He *et al.* 2025, Zhang *et al.* 2025). Empowerment outcomes accounted for 33% of coded effectiveness themes (Supplementary Figure S2 and Appendix S3).

#### Sustained engagement

Multiple studies reported continued use of digital tools following intervention, indicating potential for sustained behavioural change (Fink and Beck 2015, Lee and Kim 2019, Chang *et al.* 2021a, de Guzman and Dino 2022, Wang and Luan 2022, Miller *et al.* 2024, Yang *et al.* 2024, Yameogo *et al.* 2025). However, sustained engagement was frequently described as contingent on broader accessibility and infrastructure factors, including device availability, internet connectivity, and system usability (Fink and Beck 2015, Chang *et al.* 2021a, Wang and Luan 2022, Yang *et al.* 2024). One study (Vazquez *et al.* 2023) reported a decline in engagement after 6 months, suggesting that ongoing peer interaction and shared experiences with digital tools may help support longer-term engagement. Sustained engagement outcomes represented 19% of coded effectiveness themes (Supplementary Figure S2 and Appendix S3).

#### Positive health outcomes

Several studies reported that DHL interventions were associated with increased use of digital health tools, alongside reported improvements in health-related outcomes (Fink and Beck 2015, Nahm *et al.* 2019, Banbury *et al.* 2020, de Guzman and Dino 2020, Goransson *et al.* 2020, Pourrazavi *et al.* 2020, Bevilacqua *et al.* 2021, Chang *et al.* 2021b, Wang and Luan 2022, Vazquez *et al.* 2023, He *et al.* 2025, Zhang *et al.* 2025). These improvements were generally attributed to improved access to health information and services, as well as enhanced self-management support. Positive health outcomes accounted for 13% of coded effectiveness themes (Supplementary Figure S2 and Appendix S3).

## Discussion

This review aimed to examine how DHL interventions address the BPS dimensions in adults aged 45+ years to support meaningful engagement with digital health information. Only one study addressed all three BPS dimensions (Chang *et al.* 2021b) and reported significant improvements in DHL. However, its small sample size limits the generalizability of findings. While several other studies reported significant outcomes, none addressed all five DHL effectiveness domains (i.e. competence, critical thinking, empowerment, sustained engagement, and positive health outcomes), with most addressing three or fewer. Although Chang *et al.* (2021b) addressed most effectiveness domains, evidence of longer-term sustainability was not reported. Collectively, these findings suggest that many interventions prioritize short-term skill acquisition over deeper cognitive, behavioural, and health-related outcomes, which may limit their potential to deliver sustained benefits for older adults.

The findings indicate that adopting a BPS model may strengthen both research and practice. Psychological constructs, such as acceptance, confidence, and perceived usefulness, were the most frequently targeted components (Ahmad and Mozelius 2019, Lee and Kim 2019, Nahm *et al.* 2019, de Guzman and Dino 2020, Goransson *et al.* 2020, Pourrazavi *et al.* 2020, Bevilacqua *et al.* 2021, Chang *et al.* 2021a, 2021b, De Main *et al.* 2022, Ghorbanian Zolbin *et al.* 2022, Ngiam *et al.* 2022, Vaswani *et al.* 2023, Vazquez *et al.* 2023, Miller *et al.* 2024, Yang *et al.* 2024, He *et al.* 2025, Yameogo *et al.* 2025, Zhang *et al.* 2025). In contrast, biological and social needs were comparatively underrepresented, with only 16% of the studies addressing the biological dimension and only 27% addressing social factors. Studies addressing biological and social needs reported improvements in DHL outcomes, suggesting that integrating training addressing age-related functional constraints and the social context may support improvements in both effectiveness and sustainability outcomes of DHL interventions for older adults.

Overall, the reviewed studies most consistently reported improvements in competence and empowerment, whereas critical thinking, sustained engagement, and positive health outcomes were less frequently assessed. Most studies reported at least one positive outcome, but few addressed all five DHL effectiveness outcomes, and only one intervention addressed all BPS domains. Collectively, the review's ability to draw strong conclusions about holistic and sustained impact, highlights BPS-informed approaches as a promising direction for future research. Importantly, short-term gains in digital skills, confidence, and empowerment should be distinguished from downstream outcomes, such as sustained engagement and health improvements, for which evidence remains limited.

Methodological limitations may have influenced the strength of the evidence base. Ten of the 24 studies did not include a control group (Lee and Kim 2019, de Guzman and Dino 2020, Goransson *et al.* 2020, Bevilacqua *et al.* 2021, Li *et al.* 2021, Chang *et al.* 2021a, Miller *et al.* 2024, Yang *et al.* 2024, He *et al.* 2025), limiting confidence in causal inference (Steckler *et al.* 2020). In addition, some studies reported findings descriptively (e.g. percentages only; Malone *et al.* 2017) without accompanying inferential analyses, limiting the depth of interpretation.

Despite these limitations, studies incorporating social dimensions (e.g. perceived support) reported significant improvements (Lee and Kim 2019, Banbury *et al.* 2020, Pourrazavai *et al.* 2020, Chang *et al.* 2021b, Wang and Luan 2022, Miller *et al.* 2024, Yang *et al.* 2024, Yameogo *et al.* 2025, Zhang *et al.* 2025). These findings align with broader research demonstrating that social support reduces technology-related anxiety and promotes self-efficacy and behavioural change (Lee *et al.* 2025, Wang *et al.* 2025). Social support appears particularly important for DHL as it fosters sustained engagement and confidence. Similarly, some interventions addressing biological dimensions (Fink and Beck 2015, Ahmad and Mozelius 2019, Li *et al.* 2021, Chang *et al.* 2021b, De Main *et al.* 2022, Ghorbanian Zolbin *et al.* 2022, Ngiam *et al.* 2022, Vaswani *et al.* 2023) highlight the relevance of age-related functional changes (e.g. working memory, vision, hearing, and dexterity), which may undermine confidence, motivation, and perceived competence to engage with digital health technologies. When functional limitations intersect with psychological identity, they can directly constrain older adults’ willingness and ability to access, appraise, and use digital health information, underscoring the need for interventions that address both biological accessibility and psychological support (van Leeuwen *et al.* 2019, Mitina *et al.* 2020).

Access to healthcare may be enhanced when services incorporate tailored, user-centred components that support understanding and engagement (Mukhtar *et al.* 2025). Similarly, sustained engagement strategies (e.g. strong design, ongoing support, and behavioural change principles) are associated with more substantial and lasting improvements in health literacy (Barbati *et al.* 2025). Evidence also suggests that interventions supporting self-management, self-efficacy, and critical appraisal can enhance empowerment and health outcomes (Fitzpatrick 2023). However, while tailored, mentoring, and community-based approaches show promise (Arthanat *et al.* 2018, Betts *et al.* 2019, Marshall *et al.* 2025), a comprehensive synthesis of intervention designs, mechanisms, and outcomes remains limited. Emerging discussions further indicate that generative AI tools may support more accessible and interactive engagement with health information, particularly through conversational and personalized communication approaches (Arslan 2025). Nevertheless, concerns relating to accuracy, reliability, and the potential spread of misinformation reinforce the need for cautious, context-aware application and appropriate professional oversight (Arslan 2024, 2025).

BPS approaches have demonstrated effectiveness across healthcare contexts, including improvements in older adults’ mental health and quality of life (Chen *et al.* 2025). The BPS model has also contributed to a broader shift towards holistic, person-centred care (Engel 1977). The findings of this review reinforce the importance of addressing BPS barriers in the design and evaluation of DHL interventions for adults aged 45+ years. Interventions that focus narrowly on psychological or skills-based outcomes risk overlooking critical biological and social constraints that shape individuals’ capacity to engage meaningfully with digital health information. Given the interaction between biological changes, psychological self-concept, and social support (Engel 1977), these factors collectively influence confidence, motivation, and sustained engagement. Failure to address these interdependencies may limit both the effectiveness and durability of intervention outcomes (Granja *et al.* 2018). A BPS-informed framework therefore provides a valuable lens for identifying mediating and moderating factors, supporting a shift beyond short-term competence gains towards more holistic and sustained improvements in engagement and health-related outcomes (El Benny *et al.* 2021, Mukhtar *et al.* 2025). Integrating biological accessibility, psychological support, and social context into DHL intervention design is likely essential for achieving equitable and enduring benefits in ageing populations.

However, the BPS model has also been criticized for its limited operationalizability (Bolton and Gillett 2019). The BPS–digital model (Ahmadvand *et al.* 2018, Card 2023) extends the traditional framework by incorporating digital and technological influences as a distinct, interacting domain. In the context of DHL, this digital captures individuals’ access to, engagement with, and navigation of digital health technologies. This extension enhances contextual clarity by distinguishing outcomes from their determinants and provides a more actionable framework for intervention design, particularly in addressing system-level and technological factors (Ahmadvand *et al.* 2018, Card 2023, Gyorffy *et al.* 2023). Importantly, it strengthens the practical applicability of the model by facilitating the translation of person-centred principles into implementation and offering clearer pathways for improving intervention effectiveness.

To further address challenges in operationalizing BPS-informed approaches, the Medical Research Council (MRC) and National Institute for Health and Care Research (NIHR) framework for developing and evaluating complex interventions offers a complementary methodological scaffold (Skivington *et al.* 2021, 2024). This framework emphasizes theory-driven development, consideration of contextual and system-level influences, stakeholder engagement, and iterative testing across development, feasibility, evaluation, and implementation phases (Craig *et al.* 2008, Skivington *et al.* 2021). These principles align closely with the BPS–digital model by providing a structured mechanism for translating interacting biological, psychological, social, and digital determinants into actionable intervention components. Although no identified DHL interventions have explicitly applied the MRC/NIHR framework to date, its use in digital and mHealth interventions demonstrates its capacity to manage complexity, support mechanism-based evaluation, and enhance implementation readiness (Bobrow *et al.* 2018). Aligning DHL intervention design and evaluation with this framework may therefore improve conceptual coherence, methodological rigour, and the ability to assess sustained, multidimensional outcomes beyond short-term skill acquisition. Integrating BPS-informed theory with MRC/NIHR guidance represents a promising pathway for advancing the development, evaluation, and scalability of holistic DHL interventions for adults aged 45+ years.

### Limitations and future research

Consistent with scoping review methodology, this study employed a targeted search across five commonly used databases, which may have limited the number of eligible articles. In addition, the targeted nature of the search strategy may have introduced selectivity, as studies addressing BPS factors without explicitly using related terminology may not have been captured. Restricting inclusion to English-based publications may have further reduced the breadth and generalizability of findings. The risk-of-bias assessment indicated that nine studies were at moderate risk of bias (Fink and Beck 2015, Malone *et al.* 2017, Ahmad and Mozelurio 2019, Banbury *et al.* 2020, Bevilacqua *et al.* 2021, Wang and Luan 2022, Vaswani *et al.* 2023, Miller *et al.* 2024, He *et al.* 2025) and two at high risk (de Guzman and Dino 2020, Li *et al.* 2021). This constrains the strength of evidence base and underscores the need for more rigorous research designs and replication. Accordingly, findings should be interpreted with appropriate caution.

The findings suggest that DHL interventions for adults aged 45+ years would likely benefit from moving beyond a narrow focus on one or two BPS dimensions towards more integrative designs that explicitly address biological, psychological, and social barriers alongside digital competencies. Interventions incorporating social support and accounting for age-related functional limitations demonstrated more consistent improvements in DHL, indicating that embedding family involvement, peer learning opportunities, and accessible design features may enhance engagement, self-efficacy, and sustainability.

Future research should prioritize robust study designs with appropriate control groups, comprehensive measurement of effectiveness outcomes, and rigorous statistical analyses to strengthen the evidence base and enable meaningful comparisons across intervention types. Adopting a BPS–digital framework offers a more actionable and theoretically grounded approach by explicitly linking biological, psychological, social, and digital factors to the design and evaluation of holistic DHL interventions. Such an approach may support the development of interventions with the potential to deliver sustained benefits for older adults.

## Conclusion

This scoping review synthesizes evidence on DHL interventions for adults aged 45+ years, highlighting significant gaps in the extent to which biological, psychological, and social dimensions are addressed in an integrated manner. Most interventions prioritized short-term psychological or skills-based outcomes, particularly competence and empowerment, while comparatively few assessed biological accessibility and social context. Consequently, critical thinking, sustained engagement, and positive health outcomes (key indicators of meaningful and lasting DHL) remain inconsistently addressed and weakly evidenced. Only one intervention incorporated all three BPS dimensions, underscoring the limited translation of holistic theory into DHL practice and the constrained capacity of the current evidence base to support durable outcomes for ageing populations.

Collectively, the findings suggest that DHL interventions appear to be more effective when they move beyond narrow skill acquisition to address the interdependencies between functional capacity, psychological self-concept, and social support. Unaddressed biological constraints associated with ageing may undermine confidence and motivation, while socially supportive environments appear central to sustaining engagement and reinforcing learning. These findings reinforce the value of BPS-informed approaches and align with broader evidence highlighting the importance of holistic, person-centred models for improving health-related behaviours and outcomes in later life.

Despite this, the practical application of BPS theory within DHL research remains limited, reflecting longstanding challenges related to operationalization. The BPS–digital model offers a more actionable conceptual lens by explicitly incorporating digital determinants, while the MRC/NIHR framework for complex interventions provides a complementary methodological structure for translating theory into practice. Together, these frameworks offer a coherent pathway for designing, evaluating, and implementing DHL interventions that are theoretically grounded, context sensitive, and capable of addressing multidimensional needs across the intervention lifecycle.

In conclusion, advancing DHL for adults aged 45+ years requires a shift towards interventions that are explicitly BPS–digital in design and supported by robust methodological frameworks capable of managing complexity and assessing sustained outcomes. Future research should prioritize theory-driven, well-controlled studies that integrate biological accessibility, psychological support, and social context, alongside codesign approaches that actively involve adults aged 45+ in the development and refinement of interventions. Embedding participatory, user-informed design processes can enhance relevance and feasibility, while rigorous evaluation strategies are needed to capture long-term engagement and health-related impacts. Aligning DHL interventions with the BPS–digital model and the MRC/NIHR framework represents a promising step towards achieving equitable, effective, and enduring digital health engagement in ageing populations.

## Supplementary Material

daag080_Supplementary_Data

## Data Availability

The data underlying this article are available in Open Science Framework, at https://dx.doi.org/10.17605/OSF.IO/498GM.
