# Supplementary material for: Evaluating digital health literacy interventions for adults 45+ years: a scoping review
Source: Health Promot Int. 2026 Jun 9;41(3):daag080. doi: 10.1093/heapro/daag080 (PMC13247592; doi:10.1093/heapro/daag080)
Supplement: daag080_Supplementary_Data [file daag080_supplementary_data.zip › Appendix S1.pdf]

JBI CRITICAL APPRAISAL CHECKLIST FOR QUALITATIVE RESEARCH

Reviewer: Corresponding author Date: 5th December 2025

Author: Ahmad & Mozelius Year: 2019 DOI: https://doi.org/10.1145/3312714.3312730

	Yes	No	Unclear	Not applicable
1. Is there congruity between the stated philosophical perspective and the research methodology?	✓	☐	☐	☐
2. Is there congruity between the research methodology and the research question or objectives?	✓	☐	☐	☐
3. Is there congruity between the research methodology and the methods used to collect data?	✓	☐	☐	☐
4. Is there congruity between the research methodology and the representation and analysis of data?	✓	☐	☐	☐
5. Is there congruity between the research methodology and the interpretation of results?	✓	☐	☐	☐
6. Is there a statement locating the researcher culturally or theoretically?	☐	✓	☐	☐
7. Is the influence of the researcher on the research, and vice- versa, addressed?	☐	✓	☐	☐
8. Are participants, and their voices, adequately represented?	☐	☐	✓	☐
9. Is the research ethical according to current criteria or, for recent studies, and is there evidence of ethical approval by an appropriate body?	☐	☐	✓	☐
10. Do the conclusions drawn in the research report flow from the analysis, or interpretation, of the data?	✓	☐	☐	☐

Overall appraisal: Include ✓ Exclude ☐ Seek further info ☐

Comments (Including reason for exclusion): Although some areas such as participant voices are a little unclear, the analysis raised some good points out of the research they selected to use for the overall analysis.

JBI CRITICAL APPRAISAL CHECKLIST FOR QUALITATIVE RESEARCH

Reviewer: Corresponding author Date: 9th December 2025

Author: Vaswani et al Year: 2023 DOI: https://doi.org/10.1109/ICSE-SEIS58686.2023.00023

	Yes	No	Unclear	Not applicable
1.Is there congruity between the stated philosophical perspective and the research methodology?	✓	☐	☐	☐
2. Is there congruity between the research methodology and the research question or objectives?	✓	☐	☐	☐
3. Is there congruity between the research methodology and the methods used to collect data?	✓	☐	☐	☐
4.Is there congruity between the research methodology and the representation and analysis of data?	✓	☐	☐	☐
5.Is there congruity between the research methodology and the interpretation of results?	✓	☐	☐	☐
6.Is there a statement locating the researcher culturally or theoretically?	✓	☐	☐	☐
7.Is the influence of the researcher on the research, and vice- versa, addressed?	☐	✓	☐	☐
8.Are participants, and their voices, adequately represented?	☐	☐	✓	☐
9.Is the research ethical according to current criteria or, for recent studies, and is there evidence of ethical approval by an appropriate body?	☐	☐	✓	☐
10.Do the conclusions drawn in the research report flow from the analysis, or interpretation, of the data?	✓	☐	☐	☐

Overall appraisal: Include ✓ Exclude ☐ Seek further info ☐

Comments (Including reason for exclusion): Although some areas such as participant voices are a little unclear, further, although it states that there was ethical approval from the University, there is no evidence of this.

JBI CRITICAL APPRAISAL CHECKLIST FOR SYSTEMATIC REVIEWS AND RESEARCH SYNTHESES

Reviewer: Corresponding author Date: 5th December 2025

Author: Chang et al Year 2021 DOI: https://doi.org/10.1097/CIN.0000000000000674

	Yes	No	Unclear	Not applicable
1. Is the review question clearly and explicitly stated?	✓	☐	☐	☐
2. Were the inclusion criteria appropriate for the review question?	✓	☐	☐	☐
3. Was the search strategy appropriate?	✓	☐	☐	☐
4. Were the sources and resources used to search for studies adequate?	✓	☐	☐	☐
5. Were the criteria for appraising studies appropriate?	✓	☐	☐	☐
6. Was critical appraisal conducted by two or more reviewers independently?	✓	☐	☐	☐
7. Were there methods to minimize errors in data extraction?	✓	☐	☐	☐
8. Were the methods used to combine studies appropriate?	✓	☐	☐	☐
9. Was the likelihood of publication bias assessed?	✓	☐	☐	☐
10. Were recommendations for policy and/or practice supported by the reported data?	✓	☐	☐	☐
11. Were the specific directives for new research appropriate?	✓	☐	☐	☐

Overall appraisal: Include ✓ Exclude ☐ Seek further info ☐

Comments (Including reason for exclusion)

Good, strong systematic review

JBI CRITICAL APPRAISAL CHECKLIST FOR
SYSTEMATIC REVIEWS AND RESEARCH SYNTHESES

Reviewer: Corresponding author Date: 11th December 2025

Author: Yameogo et al DOI: https://doi.org/10.2196/69880

	Yes	No	Unclear	Not applicable
1. Is the review question clearly and explicitly stated?	✓	☐	☐	☐
2. Were the inclusion criteria appropriate for the review question?	✓	☐	☐	☐
3. Was the search strategy appropriate?	✓	☐	☐	☐
4. Were the sources and resources used to search for studies adequate?	✓	☐	☐	☐
5. Were the criteria for appraising studies appropriate?	✓	☐	☐	☐
6. Was critical appraisal conducted by two or more reviewers independently?	✓	☐	☐	☐
7. Were there methods to minimize errors in data extraction?	✓	☐	☐	☐
8. Were the methods used to combine studies appropriate?	✓	☐	☐	☐
9. Was the likelihood of publication bias assessed?	✓	☐	☐	☐
10. Were recommendations for policy and/or practice supported by the reported data?	✓	☐	☐	☐
11. Were the specific directives for new research appropriate?	✓	☐	☐	☐

Overall appraisal: Include ✓ Exclude ☐ Seek further info ☐

Comments (Including reason for exclusion)

Good, strong systematic review

JBI CRITICAL APPRAISAL CHECKLIST FOR SYSTEMATIC REVIEWS AND RESEARCH SYNTHESES

Reviewer Corresponding author Date 8th December 2025

Author Pourrazavi et al Year 2020 DOI: https://doi.org/10.1186/s13690-020-00455-6

	Yes	No	Unclear	Not applicable
1. Is the review question clearly and explicitly stated?	✓	☐	☐	☐
2. Were the inclusion criteria appropriate for the review question?	✓	☐	☐	☐
3. Was the search strategy appropriate?	✓	☐	☐	☐
4. Were the sources and resources used to search for studies adequate?	✓	☐	☐	☐
5. Were the criteria for appraising studies appropriate?	✓	☐	☐	☐
6. Was critical appraisal conducted by two or more reviewers independently?	✓	☐	☐	☐
7. Were there methods to minimize errors in data extraction?	✓	☐	☐	☐
8. Were the methods used to combine studies appropriate?	✓	☐	☐	☐
9. Was the likelihood of publication bias assessed?	✓	☐	☐	☐
10. Were recommendations for policy and/or practice supported by the reported data?	✓	☐	☐	☐
11. Were the specific directives for new research appropriate?	✓	☐	☐	☐

Overall appraisal: Include ✓ Exclude ☐ Seek further info ☐

Comments (Including reason for exclusion)

Good strong systematic review

JBI CRITICAL APPRAISAL CHECKLIST FOR
SYSTEMATIC REVIEWS AND RESEARCH SYNTHESES – SCOPING REVIEW

Reviewer Corresponding author Date 10th December 2025

Author Wang and Luan Year 2022 DOI: https://doi.org/10.3389/fpubh.2022.906089

	Yes	No	Unclear	Not applicable
1. Is the review question clearly and explicitly stated?	✓	☐	☐	☐
2. Were the inclusion criteria appropriate for the review question?	☐	☐	✓	☐
3. Was the search strategy appropriate?	✓	☐	☐	☐
4. Were the sources and resources used to search for studies adequate?	✓	☐	☐	☐
5. Were the criteria for appraising studies appropriate?	✓	☐	☐	☐
6. Was critical appraisal conducted by two or more reviewers independently?	✓	☐	☐	☐
7. Were there methods to minimize errors in data extraction?	✓	☐	☐	☐
8. Were the methods used to combine studies appropriate?	✓	☐	☐	☐
9. Was the likelihood of publication bias assessed?	☐	☐	✓	☐
10. Were recommendations for policy and/or practice supported by the reported data?	☐	☐	✓	☐
11. Were the specific directives for new research appropriate?	✓	☐	☐	☐

Overall appraisal: Include ✓ Exclude ☐ Seek further info ☐

Comments (Including reason for exclusion)

Only research question four is relevant for this review, so focus for risk assessment on this area

JBI CRITICAL APPRAISAL CHECKLIST FOR
SYSTEMATIC REVIEWS AND RESEARCH SYNTHESES: SCOPING REVIEW

Reviewer Corresponding author Date 10th December 2025

Author Yang et al Year 2024 DOI https://doi.org/10.1016/j.gerinurse.2024.07.028

	Yes	No	Unclear	Not applicable
1. Is the review question clearly and explicitly stated?	✓	☐	☐	☐
2. Were the inclusion criteria appropriate for the review question?	✓	☐	☐	☐
3. Was the search strategy appropriate?	✓	☐	☐	☐
4. Were the sources and resources used to search for studies adequate?	✓	☐	☐	☐
5. Were the criteria for appraising studies appropriate?	✓	☐	☐	☐
6. Was critical appraisal conducted by two or more reviewers independently?	✓	☐	☐	☐
7. Were there methods to minimize errors in data extraction?	✓	☐	☐	☐
8. Were the methods used to combine studies appropriate?	✓	☐	☐	☐
9. Was the likelihood of publication bias assessed?	✓	☐	☐	☐
10. Were recommendations for policy and/or practice supported by the reported data?	✓	☐	☐	☐
11. Were the specific directives for new research appropriate?	✓	☐	☐	☐

Overall appraisal: Include ✓ Exclude ☐ Seek further info ☐

Comments (Including reason for exclusion)

Good strong scoping review

JBI CRITICAL APPRAISAL CHECKLIST FOR SYSTEMATIC REVIEWS AND RESEARCH SYNTHESES

Reviewer Corresponding author Date 10th December 2025

Author Zhang et al Year 2025 DOI: https://doi.org/10.2196/66058

	Yes	No	Unclear	Not applicable
1. Is the review question clearly and explicitly stated?	✓	☐	☐	☐
2. Were the inclusion criteria appropriate for the review question?	✓	☐	☐	☐
3. Was the search strategy appropriate?	✓	☐	☐	☐
4. Were the sources and resources used to search for studies adequate?	✓	☐	☐	☐
5. Were the criteria for appraising studies appropriate?	✓	☐	☐	☐
6. Was critical appraisal conducted by two or more reviewers independently?	✓	☐	☐	☐
7. Were there methods to minimize errors in data extraction?	✓	☐	☐	☐
8. Were the methods used to combine studies appropriate?	✓	☐	☐	☐
9. Was the likelihood of publication bias assessed?	✓	☐	☐	☐
10. Were recommendations for policy and/or practice supported by the reported data?	✓	☐	☐	☐
11. Were the specific directives for new research appropriate?	✓	☐	☐	☐

Overall appraisal: Include ✓ Exclude ☐ Seek further info ☐

Comments (Including reason for exclusion)

Strong study

JBI CRITICAL APPRAISAL CHECKLIST FOR SYSTEMATIC REVIEWS AND RESEARCH SYNTHESES

Reviewer Corresponding author Date 10th December 2025

Author Zolbin et al Year 2022 DOI https://doi.org/10.1108/jd-01-2022-0004

	Yes	No	Unclear	Not applicable
1. Is the review question clearly and explicitly stated?	✓	☐	☐	☐
2. Were the inclusion criteria appropriate for the review question?	✓	☐	☐	☐
3. Was the search strategy appropriate?	✓	☐	☐	☐
4. Were the sources and resources used to search for studies adequate?	✓	☐	☐	☐
5. Were the criteria for appraising studies appropriate?	✓	☐	☐	☐
6. Was critical appraisal conducted by two or more reviewers independently?	✓	☐	☐	☐
7. Were there methods to minimize errors in data extraction?	✓	☐	☐	☐
8. Were the methods used to combine studies appropriate?	✓	☐	☐	☐
9. Was the likelihood of publication bias assessed?	✓	☐	☐	☐
10. Were recommendations for policy and/or practice supported by the reported data?	✓	☐	☐	☐
11. Were the specific directives for new research appropriate?	✓	☐	☐	☐

Overall appraisal: Include ✓ Exclude ☐ Seek further info ☐

Comments (Including reason for exclusion)

Strong study

Assessor: Corresponding author	Date of Appraisal: 5th December 2025	DOI: https://doi.org/10.1177/07334648221088281
Study Author: De Main et al	Study Title: Assessing the effects of eHe turtorials on older adults eHealth literacy	Study Year: 2022

Randomised Control study

	Internal Validity	Choice - Comments/Justification	Yes	No	Unclear	N/A
	**Bias related to selection and allocation**					
1	Was true randomization used for assignment of participants to treatment groups?	Assigned to either OnTOP or paper based NIA tutorial	☒	☐	☐	☐
2	Was allocation to treatment groups concealed?	No blinding mentions	☐	☐	☒	☐
3	Were treatment groups similar at the baseline?	Yes, demographics similar	☒	☐	☐	☐
	**Bias related to administration of intervention/exposure**					
4	Were participants blind to treatment assignment?	Not mentioned	☐	☐	☒	☐
5	Were those delivering the treatment blind to treatment assignment?	No mentioned	☐	☐	☒	☐
6	Were treatment groups treated identically other than the intervention of interest?	Same amount of training given to each group	☒	☐	☐	☐
	**Bias related to assessment, detection and measurement of the outcome**					
7	Were outcome assessors blind to treatment assignment?		Yes	No	Unclear	N/A
	OnTOP multimedia better than paper based tutorial in improving DHL	Not mentioned	☐	☐	☒	☐

8	Were outcomes measured in the same way for treatment groups?		Yes	No	Unclear	N/A
	OnTOP multimedia better than paper based tutorial in improving DHL	Both groups did Computer/internet knowledge, eHeals, Efficacy surveys, evaluation of skills surveys and procedural testing	☒	☐	☐	☐

9	Were outcomes measured in a reliable way		Yes	No	Unclear	N/A
	OnTOP multimedia better than paper based tutorial in improving DHL	Anova testing with assumptions done and no outliers found	☒	☐	☐	☐

Bias related to participant retention

10	Was follow up complete and if not, were differences between groups in terms of their follow up adequately described and analysed?					
	OnTOP multimedia better than paper based tutorial in improving DHL	Retention rate: 92%	Yes	No	Unclear	N/A
	Computer internet knowledge	All reported in tables and discussed at length in results and discussion sections	☒	☐	☐	☐
	eHealth literacy	All reported in tables and discussed at length in results and discussion sections	☒	☐	☐	☐
	Efficacy	All reported in tables and discussed at length in results and discussion sections	☒	☐	☐	☐
	Evaluation skills	All reported in tables and discussed at length in results and discussion sections	☒	☐	☐	☐
	Procedural skills	All reported in tables and discussed at length in results and discussion sections	☒	☐	☐	☐

Statistical Conclusion Validity

11	**Were participants analysed in the groups to which they were randomized?**					
	**OnTOP multimedia better than paper based tutorial in improving DHL**		**Yes**	**No**	**Unclear**	**N/A**
	Computer internet knowledge		☒	☐	☐	☐
	eHealth literacy		☒	☐	☐	☐
	Efficacy		☒	☐	☐	☐
	Evaluation skills		☒	☐	☐	☐
	Procedural skills		☒	☐	☐	☐

12	**Was appropriate statistical analysis used?**					
	**nTOP multimedia better than paper based tutorial in improving DHL**		**Yes**	**No**	**Unclear**	**N/A**
	Computer internet knowledge		☒	☐	☐	☐
	eHealth literacy		☒	☐	☐	☐
	Efficacy		☒	☐	☐	☐
	Evaluation skills		☒	☐	☐	☐
	Procedural skills		☒	☐	☐	☐

			Yes	No	Unclear	N/A
13	Was the trial design appropriate and any deviations from the standard RCT design (individual randomization, parallel groups) accounted for in the conduct and analysis of the trial?		☒	☐	☐	☐

Overall appraisal: **Include:** ☒ **Exclude:** ☐ **Seek Further Info:** ☐

Comments: No blinding could have affected outcomes.

Table 3 – The JBI Critical Appraisal Tool for RCTs

Assessor: Corresponding author	Date of Appraisal: 7th December 2025	DOI : https://doi.org/10.1177/0733464813486961
Study Author: Fink & Beck	Study Title: Developing and evaluating a web to guide older adults in their health informat searches	Study Year: 2015

Randomised Control study

	Internal Validity	Choice - Comments/Justification	Yes	No	Unclear	N/A
	**Bias related to selection and allocation**					
1	Was true randomization used for assignment of participants to treatment groups?	Used the Pew Internet using a table of random numbers	☒	☐	☐	☐
2	Was allocation to treatment groups concealed?	No clear if the researchers were 'blinded'	☐	☐	☒	☐
3	Were treatment groups similar at the baseline?		☒	☐	☐	☐
	**Bias related to administration of intervention/exposure**					
4	Were participants blind to treatment assignment?	Not mentioned	☐	☐	☒	☐
5	Were those delivering the treatment blind to treatment assignment?	No mentioned	☐	☐	☒	☐
6	Were treatment groups treated identically other than the intervention of interest?	Yes	☒	☐	☐	☐
	**Bias related to assessment, detection and measurement of the outcome**					
7	Were outcome assessors blind to treatment assignment?	It is not clear	Yes	No	Unclear	N/A

	All outcomes		☐	☐	☒	☐
8	**Were outcomes measured in the same way for treatment groups?**		**Yes**	**No**	**Unclear**	**N/A**
	Pew Internet and American life project survey		☒	☐	☐	☐
	Survey questions written by authors (not validated)		☒	☐	☐	☐
9	**Were outcomes measured in a reliable way**		**Yes**	**No**	**Unclear**	**N/A**
	Pew Internet and American life project survey	Better measures could have been done, for both outcomes, the measures were a bit vague	☐	☐	☒	☐
	Survey questions written by authors (not validated)		☐	☐	☒	☐

Bias related to participant retention

10	**Was follow up complete and if not, were differences between groups in terms of their follow up adequately described and analysed?**					
	**Internet use and experience**		**Yes**	**No**	**Unclear**	**N/A**
	Frequency of use		☒	☐	☐	☐
	How search is started		☒	☐	☐	☐
	Training made searching easier		☒	☐	☐	☐

Statistical Conclusion Validity

			Yes	No	Unclear	N/A
11	**Were participants analysed in the groups to which they were randomized?**					
	**Internet use and experience**		**Yes**	**No**	**Unclear**	**N/A**
	Frequency of use		☒	☐	☐	☐
	How search is started		☒	☐	☐	☐
	Training made searching easier		☐	☐	☐	☐

12	**Was appropriate statistical analysis used?**					
	**Internet use and experience**		**Yes**	**No**	**Unclear**	**N/A**
	Frequency of use	Although p values reported, a stronger measure could have been used, then lending itself to stronger analysis	☒	☐	☐	☐
	How search is started	Although p values reported, a stronger measure could have been used, then lending itself to stronger analysis	☒	☐	☐	☐
	Training made searching easier	Although p values reported, a stronger measure could have been used, then lending itself to stronger analysis	☒	☐	☐	☐

			Yes	No	Unclear	N/A
13	**Was the trial design appropriate and any deviations from the standard RCT design (individual randomization, parallel groups) accounted for in the conduct and analysis of the trial?**		☒	☐	☐	☐

Overall appraisal: **Include:** ☒ **Exclude:** ☐ **Seek Further Info:** ☐

Comments: Non-significant results and lack of strong measurement tools

Table 3 – The JBI Critical Appraisal Tool for RCTs

**Assessor:** Corresponding author	**Date of Appraisal: 8th December 2025**	**DOI : https://doi.org/10.1089/tmj.2018.0184**
**Study Author: Nahm et al**	**Study Title: The effects of a theory-based pat portal e-Learning program for older adults v chronic illnesses**	**Study Year: 2019**

Randomised Control study

	**Internal Validity**	**Choice - Comments/Justification**	**Yes**	**No**	**Unclear**	**N/A**
	**Bias related to selection and allocation**					
**1**	**Was true randomization used for assignment of participants to treatment groups?**	Two arm parallel group	☒	☐	☐	☐
**2**	**Was allocation to treatment groups concealed?**		☒	☐	☐	☐
**3**	**Were treatment groups similar at the baseline?**		☒	☐	☐	☐
	**Bias related to administration of intervention/exposure**					
**4**	**Were participants blind to treatment assignment?**	Not mentioned	☐	☐	☒	☐
**5**	**Were those delivering the treatment blind to treatment assignment?**	Not mentioned	☐	☐	☒	☐
**6**	**Were treatment groups treated identically other than the intervention of interest?**	Yes, control group also given access to intervention at the end of the study	☒	☐	☐	☐
	**Bias related to assessment, detection and measurement of the outcome**					

7	Were outcome assessors blind to treatment assignment?		Yes	No	Unclear	N/A
	Patient portal use	Blinding not mentioned	☐	☐	☒	☐
	Decision making		☐	☐	☒	☐
	Perceived patient provider communication		☐	☐	☒	☐
	eHeals		☐	☐	☒	☐

8	Were outcomes measured in the same way for treatment groups?		Yes	No	Unclear	N/A
	Patient portal use	Baseline/3 weeks/4 months means/SD/effect sizes	☒	☐	☐	☐
	Decision making		☒	☐	☐	☐
	Perceived patient provider communication		☒	☐	☐	☐
	eHeals		☒	☐	☐	☐

9	Were outcomes measured in a reliable way		Yes	No	Unclear	N/A
	Patient portal use		☒	☐	☐	☐
	Decision making		☒	☐	☐	☐
	Perceived patient provider communication		☒	☐	☐	☐
	eHeals		☒	☐	☐	☐

Bias related to participant retention

10	Was follow up complete and if not, were differences between groups in terms of their follow up adequately described and analysed?					
	**All outcomes**		**Yes**	**No**	**Unclear**	**N/A**
	Results	All outcomes thorough described and analysed	☒	☐	☐	☐

Statistical Conclusion Validity

11	Were participants analysed in the groups to which they were randomized?					
	**All outcomes**		**Yes**	**No**	**Unclear**	**N/A**
	Results	All participants analysed within groups	☒	☐	☐	☐

12	Was appropriate statistical analysis used?					
	**All outcomes**		**Yes**	**No**	**Unclear**	**N/A**
	Results	Full analysis: T testing, means, SD, linear mixed models, generalised for binary outcomes.	☒	☐	☐	☐

			**Yes**	**No**	**Unclear**	**N/A**
13	Was the trial design appropriate and any deviations from the standard RCT design (individual randomization, parallel groups) accounted for in the conduct and analysis of the trial?	Two arm parallel	☒	☐	☐	☐

Overall appraisal: **Include:** ☒ **Exclude:** ☐ **Seek Further Info:** ☐

Comments: Strong study

Table 3 – The JBI Critical Appraisal Tool for RCTs

RoB Assessor: Corresponding author	Date of Appraisal: 5th December 2025	DOI: https://doi.org/10.1016/j.pec.2019.10.005
Study Author: Banbury et al	Study Title: Adding value to remote monitoring: Co-design a health literacy intervention for older people with chronic disease delivered by telehealth: The telehealth literacy project	Study Year: 2019

QUASI

Internal Validity		Choice - Comments/Justification	Yes	No	Unclear	N/A
**Bias related to temporal precedence**						
1	**Is it clear in the study what is the "cause" and what is the "effect" (i.e. there is no confusion about which variable comes first)?**	Only the second question used for this scoping review, effectiveness for improving health literacy: chronic disease self-management and perception of social support.	☒	☐	☐	☐
**Bias related to selection and allocation**						
2	**Was there a control group?**	Control group were the participants who declined to be involved within the intervention.	☒	☐	☐	☐
**Bias related to confounding factors**						
3	**Were participants included in any comparisons similar?**	The control group included more females, and a lower proportion of comorbidities (4 or more chronic diseases)	☐	☒	☐	☐
**Bias related to administration of intervention/exposure**						

4	**Were the participants included in any comparisons receiving similar treatment/care, other than the exposure or intervention of interest?**		☒	☐	☐	☐

Bias related to assessment, detection and measurement of the outcome

			Yes	No	Unclear	N/A
5	**Were there multiple measurements of the outcome, both pre and post the intervention/exposure?**					
	**Effectiveness for self-management**	Health literacy questionnaire and health impact questionnaire	☒	☐	☐	☐
	**Perception of social support**	Semi structured interviews and focus groups	☒	☐	☐	☐

			Yes	No	Unclear	N/A
6	**Were the outcomes of participants included in any comparisons measured in the same way?**					
	**Effectiveness for self-management**	Control group not measured for health impact questionnaire	☐	☒	☐	☐
	**Perception of social support**	Control group not involved	☐	☒	☐	☐

			Yes	No	Unclear	N/A
7	**Were outcomes measured in a reliable way?**					
	**Effectiveness for self-management**	Anova and t-testing used – P values non-significant	☒	☐	☐	☐
	**Perception of social support**	Anova and t-testing used – P values significant	☒	☐	☐	☐

Bias related to participant retention

8	**Was follow-up complete and if not, were differences between groups in terms of their follow-up adequately described and analyzed?**					
	**Effectiveness for self-management**	Post testing done after full training after 3 months	**Yes**	**No**	**Unclear**	**N/A**
	Appraisal and active management of health information		☒	☐	☐	☐
	Engagement and navigation		☒	☐	☐	☐
	Ability to find and understand information		☒	☐	☐	☐
	**Perception of social support**		**Yes**	**No**	**Unclear**	**N/A**
	Feeling understood and supported		☒	☐	☐	☐

Statistical Conclusion Validity

9	**Was appropriate statistical analysis used?**					
	**Effectiveness of self management**	Anova and t-testing used for all outcomes	**Yes**	**No**	**Unclear**	**N/A**
	Appraisal and active management of health information		☒	☐	☐	☐
	Engagement and navigation		☒	☐	☐	☐
	Ability to find and understand information		☒	☐	☐	☐
	**Perception of social support**		**Yes**	**No**	**Unclear**	**N/A**
	Feeling understood and supported		☒	☐	☐	☐

Overall appraisal: Include: ☒ Exclude: ☐ Seek Further Info: ☐

Comments: Both groups showed small improvements in health literacy, with the intervention group gaining more—especially in perceived support from health providers and in appraising health information, though not at statistically significant levels. Qualitative findings underscored strong themes of social connection, increased confidence, reinforced health knowledge, and better self-management, with participants valuing the support, shared learning, and convenience of videoconferencing.

**RoB Assessor:** Corresponding author	**Date of Appraisal: 5th December 2025**	**DOI: https://doi.org/10.3390/ijerph182211800**
**Study Author: Bevilacqua et al**	**Study Title: eHealth Literacy: From theory to clinic application for digital health improvement. Result from the ACCESS training experience**	**Study Year: 2021**

QUASI

	Internal Validity	Choice - Comments/Justification	Yes	No	Unclear	N/A
	**Bias related to temporal precedence**					
1	**Is it clear in the study what is the "cause" and what is the "effect" (i.e. there is no confusion about which variable comes first)?**	To evaluate the new ACCESS training program	☒	☐	☐	☐
	**Bias related to selection and allocation**					
2	**Was there a control group?**	58 participants only	☐	☒	☐	☐
	**Bias related to confounding factors**					
3	**Were participants included in any comparisons similar?**	Age variation extensive 50 to 79+	☐	☒	☐	☐
	**Bias related to administration of intervention/exposure**					

4	**Were the participants included in any comparisons receiving similar treatment/care, other than the exposure or intervention of interest?**	None reported	☐	☒	☐	☐

Bias related to assessment, detection and measurement of the outcome

			Yes	No	Unclear	N/A
5	**Were there multiple measurements of the outcome, both pre and post the intervention/exposure?**					
	**eHealth literacy**	Done at pre and post (eHeals Italian version)	☒	☐	☐	☐
	**Survey of technology use**	Only done once at beginning	☐	☒	☐	☐

			Yes	No	Unclear	N/A
6	**Were the outcomes of participants included in any comparisons measured in the same way?**					
	**eHealth literacy**		☒	☐	☐	☐
	**Survey of technology use**		☒	☐	☐	☐

			Yes	No	Unclear	N/A
7	**Were outcomes measured in a reliable way?**					
	**eHealth literacy**	Frequencies used, chi square test used to evaluate	☒	☐	☐	☐
	**Survey of technology use**	Means/SD/Sapiro wilk test, comparisons done viat t-testing, chisqueare and anova	☒	☐	☐	☐

Bias related to participant retention

			Yes	No	Unclear	N/A
8	**Was follow-up complete and if not, were differences between groups in terms of their follow-up adequately described and analyzed?**					
	**eHeals**		**Yes**	**No**	**Unclear**	**N/A**
	eHeals outcomes	Full table available with statistically significant results	☒	☐	☐	☐
	**Survey of technology use (SOTU)**		**Yes**	**No**	**Unclear**	**N/A**
	SOTU	Negative, neutral, positive and totals reported	☒	☐	☐	☐

Statistical Conclusion Validity

9	**Was appropriate statistical analysis used?**					
	**eheals**		**Yes**	**No**	**Unclear**	**N/A**
	eheals	Frequencies used and percentages reported, chi square testing done for p values	☒	☐	☐	☐
	Survey of technology use		**Yes**	**No**	**Unclear**	**N/A**
	Survey of technology use	Means/SD/Sapiro wilk test, comparisons done viat t-testing, chi square and Anova	☒	☐	☐	☐

Overall appraisal: **Include:** ☒ **Exclude:** ☐ **Seek Further Info:** ☐

Comments: NO control group. Participants showed significant gains in eHealth literacy across all eHEALS items, with overall scores increasing from course start to finish. Training satisfaction was high, closely associated with higher eHealth literacy outcomes, and those willing to pay more were also more satisfied. Although age groups did not differ in perceptions of technology use (SOTU), a notable pattern emerged: participants with higher eHealth literacy reported lower positive or overall SOTU ratings. This inverse relationship suggests that as participants became more skilled in evaluating online health information, they may have adopted a more critical or discerning view of the technology.

RoB Assessor: Corresponding author	Date of Appraisal: 5th December 2025	DOI: https://doi.org/10.1016/j.gerinurse.2020.10.002
Study Author: Chang et al	Study Title: Internet health information education older adults: A pilot study	Study Year: 2021

QUASI

	Internal Validity	Choice - Comments/Justification	Yes	No	Unclear	N/A
**Bias related to temporal precedence**						
1	Is it clear in the study what is the "cause" and what is the "effect" (i.e. there is no confusion about which variable comes first)?	Although it is clear that the intervention can enhance various skills and motivations, there is only an aim, no full research question	☒	☐	☐	☐
**Bias related to selection and allocation**						
2	Was there a control group?	Testing intervention only	☐	☒	☐	☐
**Bias related to confounding factors**						
3	Were participants included in any comparisons similar?	Seven of the eleven participants already had six or more years using the internet	☒	☐	☐	☐
**Bias related to administration of intervention/exposure**						
4	Were the participants included in any comparisons receiving similar treatment/care, other than the exposure or intervention of interest?		☐	☒	☐	☐

	Bias related to assessment, detection and measurement of the outcome		Yes	No	Unclear	N/A
5	Were there multiple measurements of the outcome, both pre and post the intervention/exposure?					

			Yes	No	Unclear	N/A
	**Computer/web knowledge**	Computer web knowledge questionaire	☒	☐	☐	☐
	**Perceived usefulness/ease and enjoyment**	Korean version of the Technology acceptance model	☒	☐	☐	☐
	**Attitude towards online health information**	5 item likert scale	☒	☐	☐	☐
	**Skills and efficacy**	Korean version of eHeals	☒	☐	☐	☐
	**Understanding and applying health information**	Survey	☒	☐	☐	☐

6	**Were the outcomes of participants included in any comparisons measured in the same way?**		**Yes**	**No**	**Unclear**	**N/A**
	**Computer/web knowledge**		☒	☐	☐	☐
	**Perceived usefulness/ease and enjoyment**		☒	☐	☐	☐
	**Attitude towards online health information**		☒	☐	☐	☐
	**Skills and efficacy**		☒	☐	☐	☐
	**Understanding and applying health information**		☒	☐	☐	☐

7	**Were outcomes measured in a reliable way?**		**Yes**	**No**	**Unclear**	**N/A**
	**Computer/web knowledge**	P-value significance	☒	☐	☐	☐
	**Perceived usefulness/ease and enjoyment**	P-value significance	☒	☐	☐	☐
	**Attitude towards online health information**	P-value significance	☒	☐	☐	☐
	**Skills and efficacy**	P-value significance	☒	☐	☐	☐
	**Understanding and applying health information**	P-value significance	☒	☐	☐	☐

Bias related to participant retention

			Yes	No	Unclear	N/A
8	**Was follow-up complete and if not, were differences between groups in terms of their follow-up adequately described and analyzed?**					
	**Computer/web knowledge**		**Yes**	**No**	**Unclear**	**N/A**
	Outcome	Significant pre-post test and 2 months after the intervention	☒	☐	☐	☐
	**Perceived usefulness/ease and enjoyment**		**Yes**	**No**	**Unclear**	**N/A**
	Outcome	Non-significant pre-post test	☒	☐	☐	☐
	**Attitude towards online health information**		**Yes**	**No**	**Unclear**	**N/A**
	Outcome	Significant pre-post test and 2 months after the intervention	☒	☐	☐	☐
	**Skills and efficacy**		**Yes**	**No**	**Unclear**	**N/A**
	Outcome	Significant pre-post test and 2 months after the intervention	☒	☐	☐	☐
	**Understanding and applying health information**		**Yes**	**No**	**Unclear**	**N/A**
	Outcome	Significant pre-post test and 2 months after the intervention	☒	☐	☐	☐

Statistical Conclusion Validity

			Yes	No	Unclear	N/A
9	**Was appropriate statistical analysis used?**					
	**Computer web knowledge**		**Yes**	**No**	**Unclear**	**N/A**
	Analysis	Generalised estimating equation use with means and SD	☒	☐	☐	☐

**Perceived usefulness/ease and enjoyment**		**Yes**	**No**	**Unclear**	**N/A**
Analysis	Generalised estimating equation use with means and SD	☒	☐	☐	☐
**Attitude towards online health information**		**Yes**	**No**	**Unclear**	**N/A**
Analysis	Generalised estimating equation use with means and SD	☒	☐	☐	☐
**Skills and efficacy**		**Yes**	**No**	**Unclear**	**N/A**
Analysis	Generalised estimating equation use with means and SD	☒	☐	☐	☐
**Understanding and applying health information**		**Yes**	**No**	**Unclear**	**N/A**
Analysis	Generalised estimating equation use with means and SD	☐	☐	☐	☐

Overall appraisal: **Include:** ☒ **Exclude:** ☐ **Seek Further Info:** ☐

Comments: The program was highly feasible, consistently delivered, and achieved full session completion with minimal attrition. Participants actively engaged in guided practice, improved their computer and web-search skills, and sustained these gains for two months with the help of large-print take-home materials. Preliminary results showed significant improvements in computer/web knowledge, eHealth literacy (eHEALS), search performance, understanding of online health information, and attitudes toward internet-based health resources. While measurable behaviour changes were not observed, most participants reported adopting healthier practices based on credible online information.

**RoB Assessor:** Corresponding author	**Date of Appraisal: 5th December 2025**	**DOI: https://doi.org/10.1080/03601277.2020.1715589**
**Study Author: de Guzman & Dino**	**Study Title: Examining the role of Filipino elderly attitudes toward computer and internet on their bahavioural intention for telehealth participation.**	**Study Year: 2020**

QUASI

Internal Validity		Choice - Comments/Justification	Yes	No	Unclear	N/A
**Bias related to temporal precedence**						
1	**Is it clear in the study what is the "cause" and what is the "effect" (i.e. there is no confusion about which variable comes first)?**	Computer training will increase attitudes towards computers and the internet, positively associated with intention to participate with telehealth sessions	☒	☐	☐	☐
**Bias related to selection and allocation**						
2	**Was there a control group?**		☐	☒	☐	☐
**Bias related to confounding factors**						
3	**Were participants included in any comparisons similar?**	No demographic information given	☐	☐	☒	☐
**Bias related to administration of intervention/exposure**						
4	**Were the participants included in any comparisons receiving similar treatment/care, other than the exposure or intervention of interest?**	No demographic information given	☐	☐	☒	☐

**Bias related to assessment, detection and measurement of the outcome**						
5	**Were there multiple measurements of the outcome, both pre and post the intervention/exposure?**		**Yes**	**No**	**Unclear**	**N/A**
	**Positive intention to participate in telehealth sessions**	There was no pre testing done prior to completing the training	☐	☒	☐	☐

6	**Were the outcomes of participants included in any comparisons measured in the same way?**		**Yes**	**No**	**Unclear**	**N/A**

			Yes	No	Unclear	N/A
	**Positive intention to participate in telehealth sessions**	Some completed the attitude toward the internet scale instead of the web/internet scale, but all results were reported as one (no breakdown given)	☐	☒	☐	☐

7	**Were outcomes measured in a reliable way?**		**Yes**	**No**	**Unclear**	**N/A**
	**Positive intention to participate in telehealth sessions**	The web/internet attitude scale and the internet scale, and the computer attitude scale	☒	☐	☐	☐

Bias related to participant retention

8	**Was follow-up complete and if not, were differences between groups in terms of their follow-up adequately described and analyzed?**					
	**Positive intention to participate in telehealth sessions**		**Yes**	**No**	**Unclear**	**N/A**
	Usefulness	P values significant and given	☒	☐	☐	☐
	Liking	P values significant and given	☒	☐	☐	☐
	Confidence	P values significant and given	☒	☐	☐	☐
	Serenity	P values significant and given	☒	☐	☐	☐

Statistical Conclusion Validity

9	**Was appropriate statistical analysis used?**					
	**Positive intention to participate in telehealth sessions**		**Yes**	**No**	**Unclear**	**N/A**
	Usefulness	Without pre-test, it is impossible to understand if the intervention made a difference or not.	☐	☒	☐	☐

Serenity	Without pre-test, it is impossible to understand if the intervention made a difference or not.	☐	☒	☐	☐
Liking	Without pre-test, it is impossible to understand if the intervention made a difference or not.	☐	☒	☐	☐
Confidence	Without pre-test, it is impossible to understand if the intervention made a difference or not.	☐	☒	☐	☐

Overall appraisal: **Include:** ☒ **Exclude:** ☐ **Seek Further Info:** ☐

Comments:
No demographic info e.g., mean age, lack of information on what the intervention actually was, just states: 'basic computer and internet training for Telehealth program in a nearby state college. No control group

**RoB Assessor:** Corresponding author	**Date of Appraisal: 6th December 2025**	**DOI : https://doi.org/10.1186/s12911-020-01246-3**
**Study Author: Goransson et al**	**Study Title: An app for supporting older people receiving home care - usage, aspects of health and health literacy: A quasi-experimental study**	**Study Year: 2020**

QUASI

**Internal Validity**		**Choice - Comments/Justification**	**Yes**	**No**	**Unclear**	**N/A**
**Bias related to temporal precedence**						
1	**Is it clear in the study what is the "cause" and what is the "effect" (i.e. there is no confusion about which variable comes first)?**	Aim to describe older peoples usage of an app and evaluate the impact of the usage on health and health literacy over time.	☒	☐	☐	☐
**Bias related to selection and allocation**						

2	**Was there a control group?**	Intervention group only	☐	☒	☐	☐

Bias related to confounding factors

3	**Were participants included in any comparisons similar?**	65% were female, otherwise, similar	☒	☐	☐	☐

Bias related to administration of intervention/exposure

4	**Were the participants included in any comparisons receiving similar treatment/care, other than the exposure or intervention of interest?**	Not mentioned	☐	☐	☒	☐

Bias related to assessment, detection and measurement of the outcome

5	**Were there multiple measurements of the outcome, both pre and post the intervention/exposure?**	Baseline, end of interventions and 6 month follow up	**Yes**	**No**	**Unclear**	**N/A**
	**Swedish functional health literacy**		☒	☐	☐	☐
	**Sense of Coherence scale**		☒	☐	☐	☐
	**Commuicative and critical health literacy**		☒	☐	☐	☐

6	**Were the outcomes of participants included in any comparisons measured in the same way?**		**Yes**	**No**	**Unclear**	**N/A**
	**Swedish functional health literacy**		☒	☐	☐	☐
	**Sense of Coherence scale**		☒	☐	☐	☐
	**Commuicative and critical health literacy**		☒	☐	☐	☐

7	Were outcomes measured in a reliable way?	Reporting of P values, Wilcoxon signed rant test for ordinal and McNemars test for nominal	Yes	No	Unclear	N/A
	Swedish functional health literacy		☒	☐	☐	☐
	Sense of Coherence scale		☒	☐	☐	☐
	Communicative and critical health literacy		☒	☐	☐	☐

Bias related to participant retention

8	Was follow-up complete and if not, were differences between groups in terms of their follow-up adequately described and analyzed?					
	Swedish functional health literacy		Yes	No	Unclear	N/A
	Outcome	100% retention rate	☒	☐	☐	☐
	Sense of Coherence scale		Yes	No	Unclear	N/A
	Outcome	100% retention rate	☒	☐	☐	☐
	Communicative and critical health literacy		Yes	No	Unclear	N/A
	Outcome	100% retention rate	☒	☐	☐	☐

Statistical Conclusion Validity

9	Was appropriate statistical analysis used?					
	Sense of coherence		Yes	No	Unclear	N/A
	Analysis	Non parametric testing used. Friedman test for ordinal and Cochrans q test for nominal	☒	☐	☐	☐

Communicative and critical health literacy		Yes	No	Unclear	N/A
Analysis	Non parametric testing used. Friedman test for ordinal and Cochrans q test for nominal	☒	☐	☐	☐
**Swedish functional health literacy**		**Yes**	**No**	**Unclear**	**N/A**
Analysis	Non parametric testing used. Friedman test for ordinal and Cochrans q test for nominal	☒	☐	☐	☐

Overall appraisal: **Include:** ☒ **Exclude:** ☐ **Seek Further Info:** ☐

Comments: Training was only done via printouts/screenshots. Older adults used the Interaktor app very actively (96% median use), submitting 383 self-reports that captured 1,253 health concerns, mainly around daily/social activity limitations, pain, and fatigue. Alerts to homecare nurses led to follow-up actions, though most health outcomes showed no significant change over time. However, communicative and critical health literacy improved significantly by six months.

**RoB Assessor:** Corresponding author	**Date of Appraisal: 7th December 2025**	**DOI: https://doi.org/10.2196/69611**
**Study Author: He et al**	**Study Title: Boosting digital health engagement among older adults in Hong Kong: Pilot prepost stu of the generations connect project**	**Study Year: 2025**

QUASI

	Internal Validity	Choice - Comments/Justification	Yes	No	Unclear	N/A
	**Bias related to temporal precedence**					
1	**Is it clear in the study what is the "cause" and what is the "effect" (i.e. there is no confusion about which variable comes first)?**	Intergenerational home-based intervention package to improve digital engagement in the health space	☒	☐	☐	☐
	**Bias related to selection and allocation**					

			Yes	No	Unclear	N/A
2	Was there a control group?		☐	☒	☐	☐

Bias related to confounding factors

			Yes	No	Unclear	N/A
3	Were participants included in any comparisons similar?	Similar age group and demographics	☒	☐	☐	☐

Bias related to administration of intervention/exposure

			Yes	No	Unclear	N/A
4	Were the participants included in any comparisons receiving similar treatment/care, other than the exposure or intervention of interest?		☐	☒	☐	☐

Bias related to assessment, detection and measurement of the outcome

			Yes	No	Unclear	N/A
5	Were there multiple measurements of the outcome, both pre and post the intervention/exposure?					
	eHeals	Baseline and 2 week follow up	☒	☐	☐	☐
	Physical wellbeing	Baseline and 2 week follow up	☒	☐	☐	☐
	Screentime use	Baseline and 2 week follow up	☒	☐	☐	☐
	Exercise increase	Baseline and 2 week follow up	☒	☐	☐	☐

			Yes	No	Unclear	N/A
6	Were the outcomes of participants included in any comparisons measured in the same way?					
	eHeals		☒	☐	☐	☐
	Physical wellbeing		☒	☐	☐	☐
	Screentime use		☒	☐	☐	☐
	Exercise increase		☒	☐	☐	☐

7	**Were outcomes measured in a reliable way?**		**Yes**	**No**	**Unclear**	**N/A**
	**eHeals**	P values and Cohens d all reported	☒	☐	☐	☐
	**Physical wellbeing**	P values and Cohens d all reported	☒	☐	☐	☐
	**Screentime use**	P values and Cohens d all reported	☒	☐	☐	☐
	**Exercise increase**	P values and Cohens d all reported	☒	☐	☐	☐

Bias related to participant retention

8	**Was follow-up complete and if not, were differences between groups in terms of their follow-up adequately described and analyzed?**					
	**eHeals**		**Yes**	**No**	**Unclear**	**N/A**
	Result 1	Imputations and completed cases both reported	☒	☐	☐	☐
	Physical wellbeing		**Yes**	**No**	**Unclear**	**N/A**
	Result 1	Imputations and completed cases both reported	☒	☐	☐	☐
	**Screentime use**		**Yes**	**No**	**Unclear**	**N/A**
	Result 1	Imputations and completed cases both reported	☒	☐	☐	☐
	**Exercise increase**		**Yes**	**No**	**Unclear**	**N/A**
	Result 1	Imputations and completed cases both reported	☒	☐	☐	☐

Statistical Conclusion Validity

9	Was appropriate statistical analysis used?					
	**eHeals**		**Yes**	**No**	**Unclear**	**N/A**
	Result 1	Wilcoxon rank tests and parametric t tests	☒	☐	☐	☐
	Physical wellbeing		**Yes**	**No**	**Unclear**	**N/A**
	Result 1	Wilcoxon rank tests and parametric t tests	☐	☐	☐	☐
	**Screentime use**		**Yes**	**No**	**Unclear**	**N/A**
	Result 1	Wilcoxon rank tests and parametric t tests	☒	☐	☐	☐
	**Exercise increase**		**Yes**	**No**	**Unclear**	**N/A**
	Result 1	Wilcoxon rank tests and parametric t tests	☒	☐	☐	☐

Overall appraisal: **Include:** ☒ **Exclude:** ☐ **Seek Further Info:** ☐

Comments: Well reported and analysed.

**RoB Assessor:** Corresponding author	**Date of Appraisal: 7th December 2025**	**DOI : Lee & Kim, 2019**
**Study Author: Lee & Kim**	**Study Title: Bridging the digital divide for older adu via intergenerational mentor-up**	**Study Year: 2019**

QUASI

Internal Validity	Choice - Comments/Justification	Yes	No	Unclear	N/A

**Bias related to temporal precedence**						
1	**Is it clear in the study what is the "cause" and what is the "effect" (i.e. there is no confusion about which variable comes first)?**	Greater DHL, proactive attitudes, reduced technophobia, decrease in feelings of social isolation after training	☒	☐	☐	☐
**Bias related to selection and allocation**						
2	**Was there a control group?**		☒	☐	☐	☐
**Bias related to confounding factors**						
3	**Were participants included in any comparisons similar?**		☒	☐	☐	☐
**Bias related to administration of intervention/exposure**						
4	**Were the participants included in any comparisons receiving similar treatment/care, other than the exposure or intervention of interest?**		☐	☒	☐	☐

**Bias related to assessment, detection and measurement of the outcome**						
5	**Were there multiple measurements of the outcome, both pre and post the intervention/exposure?**		**Yes**	**No**	**Unclear**	**N/A**
	**eHealth literacy**	Pre and post test	☒	☐	☐	☐
	**Attitudes and willingness to use internet**	Pre and post test	☒	☐	☐	☐
	**Technophobia**	Pre and post test	☐	☐	☐	☐
	**Confidence**	Pre and post test	☐	☐	☐	☐
	**Social isolation**	Pre and post test	☐	☐	☐	☐

6	Were the outcomes of participants included in any comparisons measured in the same way?		Yes	No	Unclear	N/A
	eHealth literacy	T-tests, p values and effect sizes reported	☒	☐	☐	☐
	Attitudes and willingness to use internet	T-tests, p values and effect sizes reported	☒	☐	☐	☐
	Technophobia	T-tests, p values and effect sizes reported	☒	☐	☐	☐
	Confidence	T-tests, p values and effect sizes reported	☐	☐	☐	☐
	Social isolation	T-tests, p values and effect sizes reported	☐	☐	☐	☐

7	Were outcomes measured in a reliable way?		Yes	No	Unclear	N/A
	eHealth literacy	T-tests, p values and effect sizes reported	☒	☐	☐	☐
	Attitudes and willingness to use internet	T-tests, p values and effect sizes reported	☒	☐	☐	☐
	Technophobia	T-tests, p values and effect sizes reported	☒	☐	☐	☐
	Confidence	T-tests, p values and effect sizes reported	☒	☐	☐	☐
	Social isolation	T-tests, p values and effect sizes reported	☒	☐	☐	☐

Bias related to participant retention

8	Was follow-up complete and if not, were differences between groups in terms of their follow-up adequately described and analyzed?					
	**All outcomes**		**Yes**	**No**	**Unclear**	**N/A**
	Result All	All described and analysed and put into tables	☒	☐	☐	☐

Statistical Conclusion Validity

9	**Was appropriate statistical analysis used?**					
	**All outcomes**		**Yes**	**No**	**Unclear**	**N/A**
	Result All		☒	☐	☐	☐

**Overall appraisal:**	**Include:** ☒	**Exclude:** ☐	**Seek Further Info:** ☐

Comments: Well thought out study, with strong results.

**RoB Assessor:** Corresponding author	**Date of Appraisal: 7th December 2025**	**DOI : https://doi.org/10.1109/ICIM52229.2021.9417139**
**Study Author: Li et al**	**Study Title: An integrated strategy to bridge the digital divide a month the elderly: A solution base[illegible] information system**	**Study Year: 2021**

QUASI

## Internal Validity		Choice - Comments/Justification	Yes	No	Unclear	N/A
**Bias related to temporal precedence**						
1	**Is it clear in the study what is the "cause" and what is the "effect" (i.e. there is no confusion about which variable comes first)?**	Usability of a new app for seniors	☒	☐	☐	☐

Bias related to selection and allocation

2	**Was there a control group?**		☐	☒	☐	☐

Bias related to confounding factors

3	**Were participants included in any comparisons similar?**	No demographics given	☐	☐	☐	☐

Bias related to administration of intervention/exposure

4	**Were the participants included in any comparisons receiving similar treatment/care, other than the exposure or intervention of interest?**	No information regarding participants given	☐	☐	☒	☐

Bias related to assessment, detection and measurement of the outcome

			Yes	No	Unclear	N/A
5	**Were there multiple measurements of the outcome, both pre and post the intervention/exposure?**					
	**Outcome**	Only authors word that user ability and internet knowledge improved	☐	☒	☐	☐

			Yes	No	Unclear	N/A
6	**Were the outcomes of participants included in any comparisons measured in the same way?**					
	**Outcome**	Only authors word that user ability and internet knowledge improved	☐	☐	☒	☐

			Yes	No	Unclear	N/A
7	**Were outcomes measured in a reliable way?**					
	**Outcome**	Only authors word that user ability and internet knowledge improved	☐	☒	☐	☐

Bias related to participant retention

8	**Was follow-up complete and if not, were differences between groups in terms of their follow-up adequately described and analyzed?**					
	**Outcome 1**		**Yes**	**No**	**Unclear**	**N/A**
	Result	Only authors word that user ability and internet knowledge improved	☐	☒	☐	☐

Statistical Conclusion Validity

9	**Was appropriate statistical analysis used?**					
	**Outcome 1**		**Yes**	**No**	**Unclear**	**N/A**
	Result	Only authors word that user ability and internet knowledge improved	☐	☒	☐	☐

Overall appraisal: **Include:** ☒ **Exclude:** ☐ **Seek Further Info:** ☐

Comments: Although a good example of co-design, paper lacks scientific conclusions

**RoB Assessor:** Corresponding author	**Date of Appraisal: 8th December 2025**	**DOI : https://doi.org/10.1080/15398285.2017.1300040**

Study Author: Malone et al	Study Title: Perceived eHealth literacy and information behavior of older adults enrolled in a health information outreach program	Study Year: 2017

QUASI

Internal Validity		Choice - Comments/Justification	Yes	No	Unclear	N/A
**Bias related to temporal precedence**						
1	Is it clear in the study what is the "cause" and what is the "effect" (i.e. there is no confusion about which variable comes first)?	Perception and appropriate training for older adults of DHL	☒	☐	☐	☐
**Bias related to selection and allocation**						
2	Was there a control group?		☐	☒	☐	☐
**Bias related to confounding factors**						
3	Were participants included in any comparisons similar?	Only age demographic given	☐	☐	☒	☐
**Bias related to administration of intervention/exposure**						
4	Were the participants included in any comparisons receiving similar treatment/care, other than the exposure or intervention of interest?	Only age demographic given	☐	☐	☒	☐

**Bias related to assessment, detection and measurement of the outcome**						
5	Were there multiple measurements of the outcome, both pre and post the intervention/exposure?		Yes	No	Unclear	N/A
	eHeals	Only post-training tested, discussion surrounding importance for older adults.	☐	☒	☐	☐

6	Were the outcomes of participants included in any comparisons measured in the same way?		Yes	No	Unclear	N/A
	**eHeals**	All measured the same way, those that did not respond to all questions were removed from the study	☒	☐	☐	☐

7	Were outcomes measured in a reliable way?		Yes	No	Unclear	N/A
	**eHeals**	Post intervention, yes, but no comparison	☐	☐	☒	☐

Bias related to participant retention

8	Was follow-up complete and if not, were differences between groups in terms of their follow-up adequately described and analyzed?					
	**Outcome 1**		Yes	No	Unclear	N/A
	eHeals	Discussion and results unclear overall	☐	☐	☒	☐

Statistical Conclusion Validity

9	Was appropriate statistical analysis used?					
	**Outcome 1**		Yes	No	Unclear	N/A
	eHeals	Further analysis and pre intervention testing would have improved the study	☐	☒	☐	☐

Overall appraisal: **Include:** ☒ **Exclude:** ☐ **Seek Further Info:** ☐

Comments: Although this paper does not warrant being an experimental study, the perceptions of older adults warrants inclusion.

RoB Assessor: Corresponding author	Date of Appraisal: 8th December 2025	DOI : https://doi.org/10.2196/51675
Study Author: Miller et al	Study Title: Digital literacy training for low-income olders adults through undergraduate community engaged learning: Single group pretest-postest stu	Study Year: 2024

QUASI

	Internal Validity	Choice - Comments/Justification	Yes	No	Unclear	N/A
**Bias related to temporal precedence**						
1	Is it clear in the study what is the “cause” and what is the “effect” (i.e. there is no confusion about which variable comes first)?	Intergenerational training will improve older adults DHL, self-efficacy, social connectedness, and confidence	☒	☐	☐	☐
**Bias related to selection and allocation**						
2	Was there a control group?		☐	☒	☐	☐
**Bias related to confounding factors**						
3	Were participants included in any comparisons similar?	Full demographics not given	☐	☐	☒	☐
**Bias related to administration of intervention/exposure**						
4	Were the participants included in any comparisons receiving similar treatment/care, other than the exposure or intervention of interest?	Full demographics not given	☐	☐	☒	☐

Bias related to assessment, detection and measurement of the outcome

			Yes	No	Unclear	N/A
5	**Were there multiple measurements of the outcome, both pre and post the intervention/exposure?**					
	**Mobile device proficiency questionnaire**	Pre and post test	☒	☐	☐	☐
	**Loneliness**	Pre and post test	☒	☐	☐	☐
	**Attitude towards ageing**	Pre and post test	☒	☐	☐	☐

			Yes	No	Unclear	N/A
6	**Were the outcomes of participants included in any comparisons measured in the same way?**					
	**Mobile device proficiency questionnaire**	T testing and p values reported	☒	☐	☐	☐
	**Loneliness**	T testing and p values reported	☒	☐	☐	☐
	**Attitude towards ageing**	T testing and p values reported	☒	☐	☐	☐

			Yes	No	Unclear	N/A
7	**Were outcomes measured in a reliable way?**					
	**Mobile device proficiency questionnaire**	T testing and p values reported	☐	☐	☐	☐
	**Loneliness**	T testing and p values reported	☐	☐	☐	☐
	**Attitude towards ageing**	T testing and p values reported	☐	☐	☐	☐

Bias related to participant retention

8	**Was follow-up complete and if not, were differences between groups in terms of their follow-up adequately described and analyzed?**		

Mobile device proficiency questionnaire		Yes	No	Unclear	N/A
Result 1	Retention not reported	☐	☐	☒	☐
Loneliness		Yes	No	Unclear	N/A
Result 1	Retention rate not reported	☐	☐	☒	☐
**Attitude towards ageing**		Yes	No	Unclear	N/A
Result 1	Retention rate not reported	☐	☐	☒	☐

Statistical Conclusion Validity

9	**Was appropriate statistical analysis used?**					
	**Mobile device proficiency questionnaire**		**Yes**	**No**	**Unclear**	**N/A**
	Result 1	There could have been further analysis run e.g., regression analysis to further enhance the study	☐	☒	☐	☐
	**Loneliness**		**Yes**	**No**	**Unclear**	**N/A**
	Result 1	There could have been further analysis run e.g., regression analysis to further enhance the study	☐	☒	☐	☐
	**Attitude towards ageing**		**Yes**	**No**	**Unclear**	**N/A**
	Result 1	There could have been further analysis run e.g., regression analysis to further enhance the study	☐	☒	☐	☐

Overall appraisal: **Include:** ☒ **Exclude:** ☐ **Seek Further Info:** ☐

Comments: This study could have been strengthened with a control group and further analysis

RoB Assessor: Corresponding author	Date of Appraisal: 8th December 2025	DOI : https://doi.org/10.2196/40341
Study Author: Ngiam et al	Study Title: Building digital literacy in older adults low socioeconomic status in Sinapore (Project Wir Up): Non randomised controlled trial	Study Year: 2022

QUASI

	Internal Validity	Choice - Comments/Justification	Yes	No	Unclear	N/A
**Bias related to temporal precedence**						
1	Is it clear in the study what is the "cause" and what is the "effect" (i.e. there is no confusion about which variable comes first)?	To evaluate volunteer led, one-on-one home based DHL program on outcomes of loneliness, social connectedness, quality of life and wellbeing for older adults.	☒	☐	☐	☐
**Bias related to selection and allocation**						
2	Was there a control group?	Waitlist control	☒	☐	☐	☐
**Bias related to confounding factors**						
3	Were participants included in any comparisons similar?	Age, low socio economic	☒	☐	☐	☐
**Bias related to administration of intervention/exposure**						
4	Were the participants included in any comparisons receiving similar treatment/care, other than the exposure or intervention of interest?	Not mentioned	☐	☐	☒	☐

Bias related to assessment, detection and measurement of the outcome

			Yes	No	Unclear	N/A
5	**Were there multiple measurements of the outcome, both pre and post the intervention/exposure?**					
	**Loneliness**	Pre and post intervention	☒	☐	☐	☐
	**Social connectedness**	Pre and post intervention	☒	☐	☐	☐
	**Quality of life**	Pre and post intervention	☒	☐	☐	☐
	**DHL**	Pre and post intervention	☒	☐	☐	☐

			Yes	No	Unclear	N/A
6	**Were the outcomes of participants included in any comparisons measured in the same way?**					
	**Loneliness**		☒	☐	☐	☐
	**Social connectedness**		☒	☐	☐	☐
	**Quality of life**		☒	☐	☐	☐
	**DHL**		☒	☐	☐	☐

			Yes	No	Unclear	N/A
7	**Were outcomes measured in a reliable way?**					
	**Loneliness**	Regression and means tested	☒	☐	☐	☐
	**Social connectedness**	Regression and means tested	☒	☐	☐	☐
	**Quality of life**	Regression and means tested	☒	☐	☐	☐
	**DHL**	Regression and means tested	☒	☐	☐	☐

Bias related to participant retention

**8**	**Was follow-up complete and if not, were differences between groups in terms of their follow-up adequately described and analyzed?**					
	**All outcomes**		**Yes**	**No**	**Unclear**	**N/A**
	Results	All outcomes followed the transparent reporting of evaluations and nonrandomised desights reporting guidelines. Any withdrawals from the study were excluded in the results.	☒	☐	☐	☐

Statistical Conclusion Validity

**9**	**Was appropriate statistical analysis used?**					
	**All outcomes**		**Yes**	**No**	**Unclear**	**N/A**
	Results	Analysed using multiple linear regression	☒	☐	☐	☐

Overall appraisal: **Include:** ☒ **Exclude:** ☐ **Seek Further Info:** ☐

Comments: Strong study

**RoB Assessor:** Corresponding author	**Date of Appraisal: 10th December 2025**	**DOI : https://doi.org/10.2196/41809**

Study Author: Vazquez et al	Study Title: Individualistic versus collaboratoive learning in an eHealth literacy intervention for old adults: Quasi-experimental study.	Study Year: 2023

QUASI

	Internal Validity	Choice - Comments/Justification	Yes	No	Unclear	N/A
**Bias related to temporal precedence**						
1	Is it clear in the study what is the "cause" and what is the "effect" (i.e. there is no confusion about which variable comes first)?	Collaborative learning vs individualistic learning on older adults eHealth literacy	☒	☐	☐	☐
**Bias related to selection and allocation**						
2	Was there a control group?	Individualist learning	☒	☐	☐	☐
**Bias related to confounding factors**						
3	Were participants included in any comparisons similar?		☒	☐	☐	☐
**Bias related to administration of intervention/exposure**						
4	Were the participants included in any comparisons receiving similar treatment/care, other than the exposure or intervention of interest?		☒	☐	☐	☐

Bias related to assessment, detection and measurement of the outcome

			Yes	No	Unclear	N/A
5	Were there multiple measurements of the outcome, both pre and post the intervention/exposure?					
	eHeals		☒	☐	☐	☐
	Computer and web knowledge		☒	☐	☐	☐

			Yes	No	Unclear	N/A
	Basic computer and web operation		☒	☐	☐	☐
	Information seeking skills		☒	☐	☐	☐
	Website evaluation skills		☒	☐	☐	☐

6	Were the outcomes of participants included in any comparisons measured in the same way?		Yes	No	Unclear	N/A
	eHeals	3 x 2 x 3 mixed factorial design	☒	☐	☐	☐
	Computer and web knowledge	3 x 2 x 3 mixed factorial design	☒	☐	☐	☐
	Basic computer and web operation	3 x 2 x 3 mixed factorial design	☒	☐	☐	☐
	Information seeking skills	3 x 2 x 3 mixed factorial design	☒	☐	☐	☐
	Website evaluation skills	3 x 2 x 3 mixed factorial design	☒	☐	☐	☐

7	Were outcomes measured in a reliable way?		Yes	No	Unclear	N/A
	eHeals	F-test and p-values all reported, with means & SD	☒	☐	☐	☐
	Computer and web knowledge	F-test and p-values all reported, with means & SD	☒	☐	☐	☐
	Basic computer and web operation	F-test and p-values all reported, with means & SD	☒	☐	☐	☐
	Information seeking skills	F-test and p-values all reported, with means & SD	☒	☐	☐	☐
	Website evaluation skills	F-test and p-values all reported, with means & SD	☒	☐	☐	☐

Bias related to participant retention

8	**Was follow-up complete and if not, were differences between groups in terms of their follow-up adequately described and analyzed?**					
	**All outcomes**		**Yes**	**No**	**Unclear**	**N/A**
	Results	All adequately described, discussed and reported	☒	☐	☐	☐

Statistical Conclusion Validity

9	**Was appropriate statistical analysis used?**					
	**All outcomes**		**Yes**	**No**	**Unclear**	**N/A**
	Results	Factorial design	☒	☐	☐	☐

Overall appraisal: **Include:** ☒ **Exclude:** ☐ **Seek Further Info:** ☐

Comments: Strong study, well reported and evaluated.
