# Supplementary material for: Evaluating digital health literacy interventions for adults 45+ years: a scoping review
Source: Health Promot Int. 2026 Jun 9;41(3):daag080. doi: 10.1093/heapro/daag080 (PMC13247592; doi:10.1093/heapro/daag080)
Supplement: daag080_Supplementary_Data [file daag080_supplementary_data.zip › Appendix S2.docx]

BPS scoping

Codes

| Name | Description | Files |
| --- | --- | --- |
| Biological |  | 0 |
| Consideration of age-related changes |  | 6 |
| Tailored interventions to consider biological |  | 6 |
| Psychological |  | 0 |
| Behavioural change |  | 11 |
| Improved self-efficacy |  | 16 |
| Technology acceptance |  | 15 |
| Trust |  | 5 |
| Social |  | 0 |
| Feelings of connectedness |  | 11 |
| Perceived feelings of support |  | 9 |
