# Supplementary material for: Evaluating digital health literacy interventions for adults 45+ years: a scoping review
Source: Health Promot Int. 2026 Jun 9;41(3):daag080. doi: 10.1093/heapro/daag080 (PMC13247592; doi:10.1093/heapro/daag080)
Supplement: daag080_Supplementary_Data [file daag080_supplementary_data.zip › Appendix S3.docx]

| **Codes** | **Number of coding references** | **Aggregate number of coding references** | **Number of items coded** | **Aggregate number of items coded** |
| --- | --- | --- | --- | --- |
| Codes\\Competence\Co-design to enhance competence and useablitiy | 12 | 12 | 11 | 11 |
| Codes\\Competence\Intergenerational, peer or group training | 14 | 14 | 13 | 13 |
| Codes\\Competence\Self-belief | 5 | 5 | 5 | 5 |
| Codes\\Critical thinking\Evaluate | 8 | 8 | 8 | 8 |
| Codes\\Empowerment\Multimedia and group learning | 15 | 15 | 14 | 14 |
| Codes\\Empowerment\Training for independence | 22 | 22 | 18 | 18 |
| Codes\\Postive health outcomes\DHL creates | 14 | 14 | 14 | 14 |
| Codes\\Sustained engagement\Accessibility | 8 | 8 | 7 | 7 |
| Codes\\Sustained engagement\Behavioural change | 13 | 13 | 12 | 12 |
