# Supplementary material for: Evaluating digital health literacy interventions for adults 45+ years: a scoping review
Source: Health Promot Int. 2026 Jun 9;41(3):daag080. doi: 10.1093/heapro/daag080 (PMC13247592; doi:10.1093/heapro/daag080)
Supplement: daag080_Supplementary_Data [file daag080_supplementary_data.zip › Table S1.docx]

**Characteristics Table**

*Characteristics of the included studies*

| Authors | Location | Study design | Study setting | N | Age | Year of Publication | Theories & models tested |
| --- | --- | --- | --- | --- | --- | --- | --- |
| Ahmad & Mozelius | Sweden | Qualitative | N/A | Undisclosed | 65+ | 2019 | Technology Acceptance Model |
| Banbury et al | Australia | Mixed Method Quasi experimental | Aged care service delivery | 111 | 50+ | 2019 | Social ecological approach |
| Bevilacqua et al | Italy | Quasi experimental | GoToMeeting platform | 58 | 50+ | 2020 | European Qualifications Framework |
| Chang et al a | Korea | Systematic review | N/A | Undisclosed | 47+ | 2021 | Guthrie's Model for Web Searching, Technology Acceptance Model, Social Interdependence Theory, Cognitive Theory of Multimedia Learning, Knowles’ Theory of Andragogy, Comprehensive Model of Information Seeking, Information Framework, Structural Influence Model of Health Communication, Conceptual Model of Avoidance of Internet Health Information, Health Belief Model, Theory of Consumers' Preventive Health Behaviours |
| Chang et al b | Korea | Quasi experimental | Older adult friendly centres | 11 | 65+ | 2021 | Motivation-Behavioural Skills Model |
| de Guzman & Dino | Philippines | Quasi experimental | Hospital and health care centres | 82 | Senior | 2020 | Diffusion of Innovation |
| De Main et al | USA | Randomised controlled trial | Senior centres and public libraries | 91 | 65+ | 2022 | Cognitive Theory of Multimedia Learning |
| Fink & Beck | USA | Randomised controlled trial | Online | 66 | 50+ | 2015 | Knowles Theory |
| Goransson et al | Sweden | Quasi experimental | Home care | 17 | 65+ | 2020 | Participation and person-centered care framework |
| He et al | China | Quasi experimental | Home visits and group sessions | 101 | 65+ | 2025 | Information-Motivation-Behavioural Skills Model |
| Lee & Kim | USA | Quasi experimental | Senior centres | 55 | 65+ | 2019 | Knowles Theory |
| Li et al | China | Quasi experimental | Unknown | 10 | Senior | 2021 | None |
| Malone et al | USA | Quasi experimental | Library | 30 | Seniors | 2017 | None |
| Miller et al | USA | Quasi experimental | Senior centre | 18 | 60+ | 2024 | Senior Technology Acceptance and Adoption Model combined with Center for Research and Education on Ageing and Technology Enhancement Model |
| Nahm et al | USA | Randomised control trial | Online | 239 | 50+ | 2019 | Self-Efficacy Theory |
| Ngiam et al | Singapore | Quasi experimental | Home-based | 138 | 55+ | 2022 | None |
| Pourrazavi et al | Iran | Systematic review | N/A | 1417 | 60+ | 2020 | Health Belief Model, Self-Efficacy Theory, Technology Acceptance Model, Diffusion of Innovations Model, Social Interdependence Theory, Cognitive Theory of Multimedia Learning |
| Vaswani et al | UK | Qualitative | Unknown | 10 | 74+ | 2023 | Social Cognitive Theory, Knowles Theory |
| Vazquez et al | USA | Quasi experimental | Senior activity centres | 466 | 60+ | 2023 | None |
| Wang & Luan | China | Scoping review | N/A | 848 | Seniors | 2022 | Health Belief Model, Health Decision Making Self-Efficacy, Digcomp, Information-Motivation-Behavioural Skills Model, |
| Yameogo et al | Canada | Systematic review | N/A | 355 | Seniors | 2025 | Social Learning Approach |
| Yang et al | China | Scoping review | N/A | 3404 | 60+ | 2024 | Self-Efficacy Theory, Social Interdependence Theory, Health Belief Model, Knowles Theory, Digcomp, Cognitive Theory of Multimedia Learning, Information-Motivation-Behavioural Skills Model, Self-Determination Theory, Intervention Mapping |
| Zhang et al | Malaysia | Systematic review | N/A | Undisclosed | 50+ | 2025 | Social Media Health Behaviours, Social Ecological Model |
| Zolbin et al | Finland | Systematic review | N/A | Undisclosed | 65+ | 2022 | None |
