# Supplementary material for: Evaluating digital health literacy interventions for adults 45+ years: a scoping review
Source: Health Promot Int. 2026 Jun 9;41(3):daag080. doi: 10.1093/heapro/daag080 (PMC13247592; doi:10.1093/heapro/daag080)
Supplement: daag080_Supplementary_Data [file daag080_supplementary_data.zip › Table S2.docx]

**Interventions and Measures**

*Interventions and measures used*

| Authors | Intervention | Measures | Results |
| --- | --- | --- | --- |
| Ahmad & Mozelius | Formative evaluation | Thematic analysis | Trust, privacy, technology acceptance, and social support identified |
| Banbury et al. | Five weekly 1.5-h videoconference sessions vs remote monitoring | Health Literacy Questionnaire; Health Impact Education Questionnaire | Significant improvements across two domains (i.e., appraisal of health information, actively managing health; *p* < .02) |
| Bevilacqua et al. | Four-week blended, interactive training | eHEALS (ehealth literacy scale); Technology Use Survey | eHEALS increased from pre- to post-intervention (*p* = .001) |
| Chang et al. (a) | Systematic review | Frameworks, factors, outcomes | Improvements in eHL efficacy, knowledge, recall, evaluation, and performance |
| Chang et al. (b) | Five weekly 2-h computer and internet skills classes | eHEALS; TAM; attitudes; author-developed tools | All outcomes significant (*p* ≤ .002) |
| de Guzman & Dino | Computer and internet training for telehealth | Attitude and intention scales | Confidence, usefulness, liking, and serenity significant (*p* < .02) |
| De Main et al. | Multimedia vs. paper-based tutorial (4 sessions/2 weeks) | Computer knowledge; eHEALS | No significant group differences between multimedia and paper tutorial training, but both resulted in significant results in self-efficacy, knowledge and attitudes towards computer use. |
| Fink & Beck | Guided searches vs. slide-based tutorial (70 min) | Author survey (knowledge, self-efficacy) | No significant differences between guided searches and slide-based tutorial, but both improved knowledge and self-efficacy for participants |
| Göransson et al. | Interaktor app use (3 months) | Functional and communicative HL scales | Communicative and critical HL significant (*p* < .01); functional NS |
| He et al. | Nurse-led home visits + 14-day WhatsApp coaching | eHEALS; adapted health surveys | Physical wellbeing significant (*p* = .04); other measures non-significant |
| Lee & Kim | Intergenerational mentoring (6 sessions) | eHEALS; attitudes; loneliness | All outcomes significant (*p* < .01) |
| Li et al. | WeChat and online tutorial | None | No quantitative results reported, only authors commentary |
| Malone et al. | Twenty-four-session NIA toolkit program | eHEALS | Descriptive results only; scale under development |
| Miller et al. | Eight-week student-led training with device provision | Mobile proficiency; attitudes | Proficiency (*p* < .001); ageing attitude (*p* = .01) |
| Nahm et al. | Three-week T-PeP modules vs control | eHEALS; self-efficacy scales | eHEALS (*p* = .01); communication/self-efficacy (*p* ≤ .004) |
| Ngiam et al. | Project Wire Up (six sessions/3 months) | Digital literacy; social scales | Digital literacy significant (*p* < .001) |
| Pourrazavi et al. | Systematic review | Theory-based synthesis | Self-efficacy frameworks most effective |
| Vaswani et al. | Elderlearn intergenerational prototype | Thematic analysis | Positive understanding, navigation, and intention |
| Vazquez et al. | Eight-session individual vs collaborative learning | eHL efficacy; skills measures | Post-test gains significant (*p* < .001); declined at 6 months |
| Wang & Luan | Review of DHL methods | Narrative synthesis | Face-to-face and online programs effective but limited |
| Yameogo et al. | Peer-guided community-based learning | Social practice analysis | Improved autonomy and social participation |
| Yang et al. | Review of intervention effects | Narrative synthesis | Hybrid online–offline approaches most effective |
| Zhang et al. | Review of social media and eHL | Narrative synthesis | Social media training enhances eHL with sustained support |
| Zolbin et al. | Review of HL and digital services | Thematic synthesis | Improved HL skills, management competency, and confidence |

*Note****.*** *eHL = eHealth literacy; HL = health literacy; NS = not significant; TAM = Technology Acceptance Model; T-PeP = Theory-Based Patient Portal program*
