# Supplementary figures and images for: Evaluating digital health literacy interventions for adults 45+ years: a scoping review
Source: Health Promot Int. 2026 Jun 9;41(3):daag080. doi: 10.1093/heapro/daag080 (PMC13247592; doi:10.1093/heapro/daag080)

**Figure 2**

*Themes for Biopsychosocial Integration in Training*

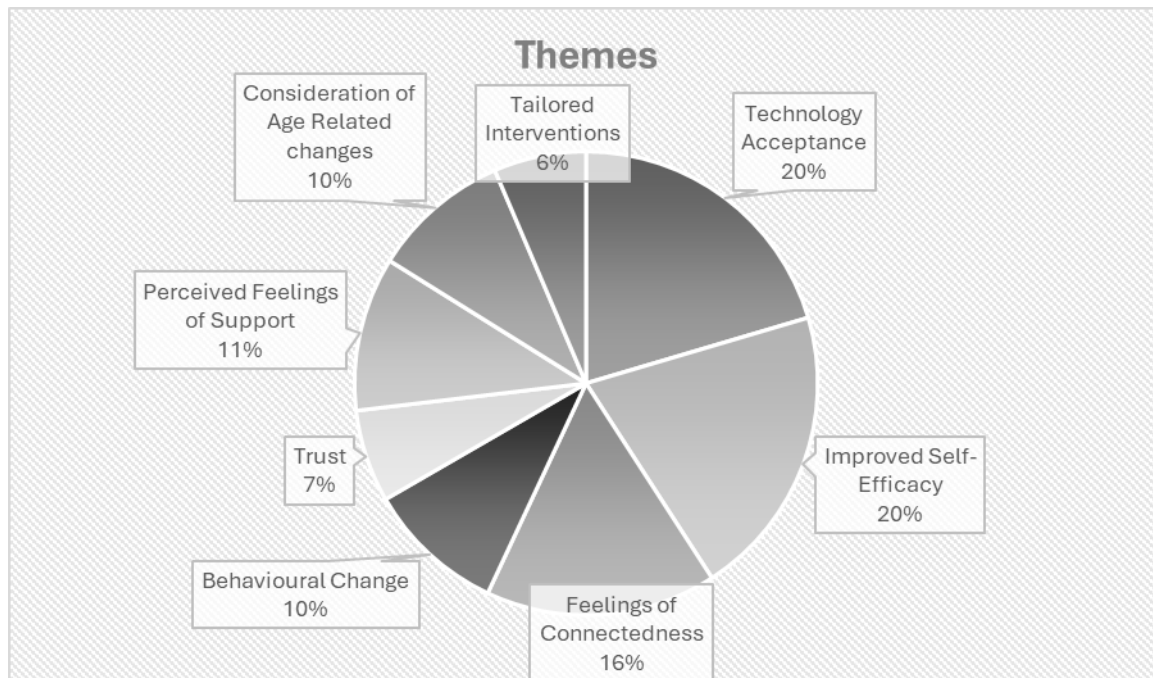

Supplement: daag080_Supplementary_Data [file daag080_supplementary_data.zip › Figure S1.pdf]

**Figure 3**

*Themes for Effectiveness of Training*

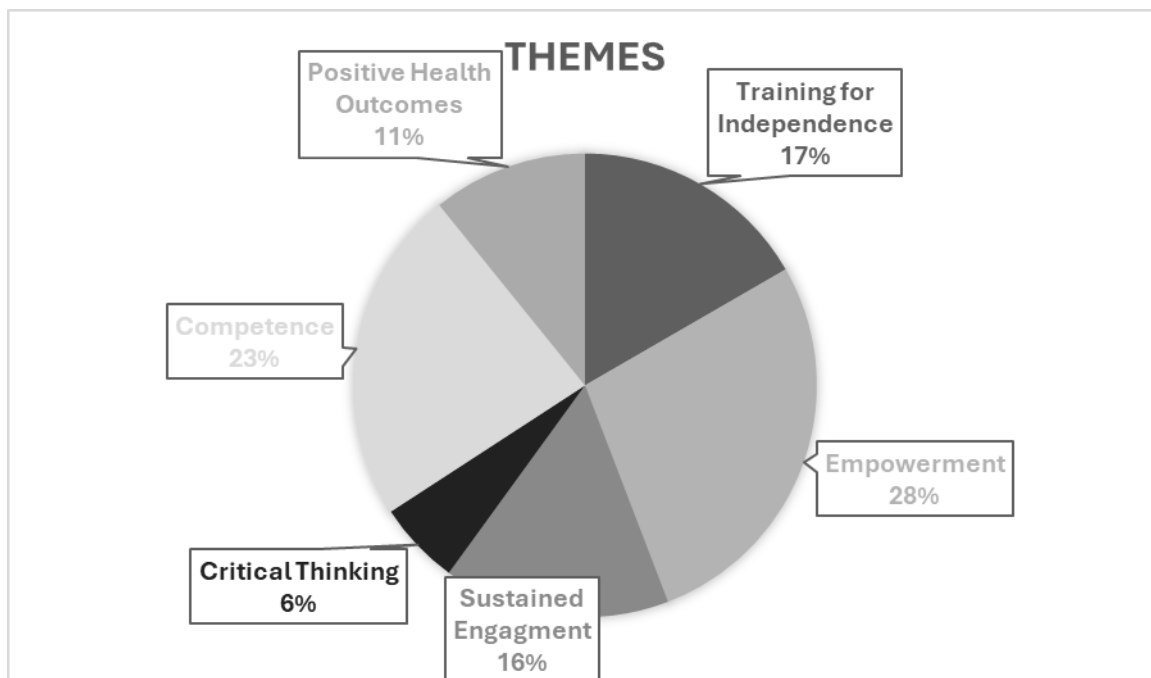

Supplement: daag080_Supplementary_Data [file daag080_supplementary_data.zip › Figure S2.pdf]
